# Evaluation of Anti-Inflammatory, Antidiabetic, Antioxidant, and Anticholinergic Activities, as Well as Chemical Composition and Polyphenolic Compounds in Novel SCOBY-Fermented Juices

**DOI:** 10.3390/molecules30091940

**Published:** 2025-04-27

**Authors:** Joanna Grondalska, Joanna Kolniak-Ostek

**Affiliations:** Department of Fruit, Vegetable and Plant Nutraceutical Technology, Wrocław University of Environmental and Life Sciences, 37 Chelmonskiego Street, 51-630 Wroclaw, Poland; 121382@student.upwr.edu.pl

**Keywords:** SCOBY, apple juice, pear juice, anti-inflammatory activity, antioxidant capacity, antidiabetic activity, anticholinergic properties, UPLC/DAD/qTOF-MS/MS

## Abstract

Fermentation processes, which occur under the influence of multiplying microorganisms, lead to the creation of products with beneficial health properties. Due to the growing interest of consumers in beverages with health-promoting properties, new raw materials and their processing methods are being intensively studied to obtain products with improved functional values. The purpose of the study is to determine the effect of fermentation using SCOBY (Symbiotic Culture of Bacteria and Yeast) on the chemical composition, polyphenolic profile, and biological activity of apple and pear juices. The fermentation process caused a decrease in the content of polyphenols in apple juice from 1568.8 to 1269.0 mg/L, while in pears, an increase was observed from 492.9 to 576.7 mg/L. Statistically significant changes were observed in the content of individual groups of polyphenolic compounds. The fermentation process also influenced the increase in the value of anti-inflammatory, antioxidant, antidiabetic, and anticholinergic activity. This indicates that fermentation can be an effective process in increasing the biological properties of fruit juices. This fact can be used in the prevention of lifestyle diseases and in the production of functional foods with targeted health-promoting properties.

## 1. Introduction

Modern research emphasizes the key importance of fruits and vegetables as basic elements of a balanced diet, providing essential vitamins, minerals, sugars, organic acids, fiber, and pectins. According to WHO guidelines, the recommended daily intake of these products is approximately 400 g (that is, five portions), one of which can be replaced by a glass of juice [1]. Fruit juices, valued for their attractive taste, smell, and the presence of bioactive compounds, are an interesting area of research on their potential to protect against oxidative stress [1]. In particular, apples, being a rich source of polyphenols—including flavonols such as quercetin and its glycosides and anthocyanins—show health-promoting properties [2], while pears, due to the presence of compounds with strong antioxidant and anti-inflammatory effects, shape their antioxidant potential [3]. Including fruit juices in the daily diet might contribute to the prevention of chronic diseases, such as circulatory system diseases, cancer, and neurodegenerative diseases [4], and also support the prevention of many lifestyle diseases.

Noncommunicable diseases (NCDs), which occur worldwide, are mainly associated with a poor lifestyle, including poor nutrition, low physical activity, chronic stress, environmental pollution, industrialization, noise, and the use of stimulants [5,6]. This group of diseases includes, among others, obesity, hypertension, diabetes, circulatory system diseases (including coronary heart disease and myocardial infarction), atherosclerosis, osteoporosis, and cancers [5,6]. Prevention of these diseases is based on an integrated approach, including physical activity, stress reduction, and avoidance of addictions [7], as well as the use of a diet rich in nutrients, including polyphenolic compounds with documented antioxidant, anti-inflammatory, anticancer, and antimicrobial effects [5].

Fermentation, as one of the oldest methods of food preservation, has been used for thousands of years to extend shelf life and modify taste and nutritional value. Fermentation processes, which occur under the influence of multiplying microorganisms, lead to the creation of products with beneficial health properties [8]. An example of the application of this technology is the production of kombucha, a fermented drink obtained mainly from black tea and sucrose, with the participation of symbiotic cultures of yeast and acetic acid bacteria (AAB) and occasionally lactic acid bacteria (LAB), embedded in a cellulose membrane [8,9]. Kombucha fermentation, occurring on the liquid surface, leads to the gradual formation of successive layers of cellulose and the generation of compounds such as ethyl alcohol, organic acids (acetic, lactic, gluconic), ascorbic acid, vitamin D, thiamine, caffeine, sugars, and enzymes [10,11]. Numerous scientific studies have shown that moderate consumption of kombucha may have probiotic, anti-inflammatory, antibacterial, anticancer, antioxidant (immune support), anticholinergic, and antidiabetic effects, as well as contribute to lowering cholesterol levels and supporting the liver detoxification process [9,10,12,13].

Due to the growing interest of consumers in beverages with health-promoting properties, new raw materials and their processing methods are being intensively studied to obtain products with improved functional values [14]. Despite the dynamic development of this area, the number of studies on fermented juices remains limited, indicating the need for further and in-depth analyses. Studies using new substrates suggest that the fermentation process can be faster compared to traditional methods of producing kombucha, which is strongly dependent on the sugar content (sucrose, glucose, fructose) in the raw material [14]. The key aspect of this process is the effective interaction between the substrate and the microorganism culture, which enables the decomposition of sugars into organic acids by acetic acid bacteria while at the same time enabling synergy with yeast, which produces ethanol, which is used by bacteria for the subsequent production of acetic acid. In addition, yeast autolysis results in the release of vitamins, acting as natural stimulants for microorganism growth [14].

In relation to the above data, this article analyses both the importance of nutrients present in fruit juices and the potential of fermentation processes conducted using SCOBY (Symbiotic Culture of Bacteria and Yeast) in the production of beverages with health-promoting properties, which is the basis for developing new strategies for the prevention of lifestyle diseases. The study was carried out in apple and pear juices, which were subjected to a 14-day fermentation. The influence of the fermentation process on changes in chemical composition (pH, extract, sugar content, organic acids, and polyphenols) and biological properties, such as anti-inflammatory, antidiabetic, antioxidant, and anticholergenic properties, was determined in juices.

## 2. Results and Discussion

### 2.1. Basic Chemical Composition

#### 2.1.1. pH and Total Soluble Solids

One of the most important parameters controlled during the entire fermentation process is pH. Changes indicate biochemical processes occurring, which are the result of the activity of fermentation microorganisms. The organic acids produced cause a decrease in pH and ensure microbiological safety by limiting the multiplication of pathogenic microorganisms that cannot develop under such conditions. According to the regulations, for a kombucha-type drink to be considered safe, its pH must be in the range of 2.5 to 4.2 [8,15]. Values higher than in the given range may increase the risk of developing undesirable microorganisms, while lower values indicate too high acetic acid levels [16].

During the fermentation process of the fruit juices in both variants, a decrease in pH was observed (Table 1).

A comparison of the initial pH value with the final one showed that pear juice showed a greater decrease in pH compared to apple juice, indicating a more intensive fermentation process and a greater amount of organic acids produced. On the other hand, apple juice was characterized by a lower pH on the last day of fermentation than pear juice. The pH of apple juice decreased by 2.82%—from 3.19 to 3.10, while in the pear juice sample, a decrease in the pH value by 19.42% was observed—from pH 4.12 to 3.32. During the entire fermentation process, the pH of the juices tested was not exceeded and was within the permissible range (Table 1).

Morales et al. [16] performed analyses of kombuchas from freeze-dried fruits such as cherry, plum, apricot, strawberry, persimmon, grape, orange, and pomegranate. Before fermentation, the pH values of the fruit solutions tested ranged between 3.30 and 3.44, depending on the raw material. The highest pH values were found in samples of apricot and cherry, while the lowest was found in orange. Each sample had lower pH values compared to the tea (pH = 3.66). These differences are caused by different compositions of selected substrates. In the initial days of fermentation, the parameter tested decreased slightly and slowly, and in the following days, it decreased intensively and significantly. The final pH values after 21 days of the process were 2.03–2.75, and more important, the lowest pH values were found in persimmon kombucha, while the highest was found in grape kombucha. After analyzing the results, it was concluded that fermentation in raw materials that reached pH outside the permitted range should be stopped before the amount of acetic acid exceeded the safe range [16]. In the study by Chong et al. [17], the pH decreased from 3.19–3.27 to 2.72–2.79 on the 10th day of fermentation. It is important that the values of the tested samples are within the permitted range of 2.5 to 4.2. Fonteles et al. [18] also showed that the values decrease in the subsequent days of fermentation from 4.10 to 2.98 on the 12th day of the process. As the fermentation progresses, the pH decreases due to the organic acids produced by SCOBY [17].

The tested kombucha variants show a similar decrease in the total content of soluble solids during fermentation (Table 1), which depends on the duration of the process. Refractometric determination showed that the initial samples contained the most total soluble solids. As the fermentation process progressed, this amount decreased, reaching the lowest value on the 14th day of the process. In apple juice, the value decreased by 51.49% (from 11.75 °Brix to 5.70 °Brix), and in pear juice by 51.72% (from 11.60 °Brix to 5.60 °Brix) (Table 1).

In his work, Tomar [19] showed that kombucha prepared from dried raw materials such as black grapes, black mulberries, and rosehips showed a decrease in the Brix value during 21 days of fermentation. In a study by Jakubczyk et al. [20], the sugar content in the tea decreased from 11–10.75 °Brix to 9.5–7.5 °Brix on the 14th day of fermentation. The longer the fermentation lasted, the lower the sugar content. The decrease in total soluble solids is related to the use of sugars in the mash by microorganisms (yeasts) to produce ethanol [20]. Then, acetic acid bacteria use ethanol to produce acetic acid [19].

#### 2.1.2. Organic Acids and Reducing Sugars

During fermentation, thanks to symbiotic cultures of bacteria and yeasts located in the cellulose matrix, significant changes occur. Enzymatic decomposition of sucrose into glucose and fructose catalyzed by yeast-derived invertase occurs, generating ethanol through glycolysis [15]. Additionally, sucrose is the main carbon substrate and provides essential nutrients that condition the proper growth of microorganisms. During the processes, microorganisms produce compounds that affect the health properties and taste and smell of the final product [10].

The juices analyzed show a decrease in the content of reducing sugars (Table 1). In pear juice, glucose disappeared on the 10th day of fermentation. This indicates the complete use of sugars by microorganisms.

A study conducted by Nurikasari et al. [21] showed that the amount of sugar decreased from the 1st to the 11th day of fermentation. This confirms the fact that every microorganism needs sugar as a source of carbon, which is used as food and for further biochemical transformations. Also, observations conducted by Zubaidah et al. [22] showed that in each of the apple kombucha varieties analyzed, the total sugar content decreased during the ongoing process. The decreasing amount of glucose is caused by its use for the production and growth of new microbial cells during fermentation, and during these transformations, metabolites such as organic acids are created. Yeast, *Saccharomyces cereviseae*, uses the glucose contained in the raw material as a source of energy and decomposes it, producing ethanol and carbon dioxide [22].

Kombucha-type beverages are produced as a result of fermentation carried out by acetic acid bacteria, lactic acid bacteria, and yeast. Sucrose is metabolized under aerobic conditions by yeast, and the result of these transformations is the formation of simple sugars, organic acids, ethyl alcohol, and carbon dioxide, which undergo further aerobic biochemical reactions by acetic acid bacteria, leading to the formation of acetic acid and acetaldehyde [18]. Organic acids generated during the process result in a decrease in the pH of the produced beverage and ensure microbiological safety by inhibiting the multiplication of potentially pathogenic microorganisms that cannot grow in an acid environment. An increase in acidity may be one of the main indicators that determines the degree of fermentation of the mixture [10]. The content of organic acids can vary depending on the SCOBY starter culture, the amount of sugar present, the temperature, and the time of the process [12]. The samples tested showed that the process influenced the increase in malic acid content in both variants analyzed (Table 1). Its highest value was determined on the 12th and 14th day in apple juice and on the 6th and 12th day in pear juice. A greater amount of malic acid was formed during the fermentation of apple juice. The concentration of acetic acid in the tested samples of both variants increased, and a larger amount of the identified acid was formed in pear juice. The highest amount was observed on the 8th day of fermentation in apple juice (0.91 g 100 mL) and in pear juice on the 6th and 12th day of the process (0.40 g/100 mL). Furthermore, on the 12th day of pear juice fermentation and on the 10th day of apple juice fermentation, the formation of lactic acid was observed in the amount of 0.03 g/100 mL.

In the study by Jakubczyk et al. [23], the acetic acid content during the fermentation of kombucha prepared from four types of leaf tea (green, black, white, and red) increased. The highest acid concentration was recorded on the 14th day of fermentation for all beverages analyzed (9071.02–9147.40 mg/l). Also, in the studies by Pawluś and Kolniak-Ostek [10], it was found that the concentration of acetic and malic acid increased with the progress of fermentation in all batches tested. It was found that a larger amount of sucrose present in the samples affected the greater production of organic acids during the process.

Lactic acid bacteria, especially of the genus Lactobacillus, play a key role in the fermentative production of lactic acid (2-hydroxypropionic acid). This process involves the conversion of carbohydrates (mainly glucose) into lactic acid under anaerobic conditions. Lactobacillus bacteria, which are among those present in SCOBY, are characterized by the ability to efficiently synthesize the L(+)-lactic acid form, which is important from the point of view of pharmaceutical and biomedical applications [24]. In the Sheeladevi study [25], it was shown that optimization of culture conditions, such as temperature, pH, substrate concentration, and incubation time, significantly affects fermentation efficiency. At the same time, more and more attention is paid to sustainable and economically viable carbon sources, such as fruit pulp, as effective substrates for lactic acid biosynthesis [26,27].

The influence of malic and acetic acids on the quality of fermented products is complex and includes both sensory and microbiological aspects. Both of these organic compounds play an important role in modulating the final properties of fermented foods through their interactions with microorganisms, especially lactic acid bacteria (LAB). Malic acid, a natural component of many raw plant materials, is transformed into lactic acid during malolactic fermentation carried out by LAB. This process reduces the total acidity of the product, thus improving its microbiological stability and reducing the risk of contamination by undesirable microorganisms [28,29]. At the same time, malolactic fermentation has a positive effect on organoleptic characteristics, enriching the aromatic and flavor profile, which is used, among others, in wine production [28,29]. In turn, acetic acid, produced mainly by acetic acid bacteria, has strong antimicrobial properties, which can be beneficial from a microbiological safety point of view. However, its excessive concentrations are undesirable, as they lead to a deterioration of the sensory qualities of the product—especially in wine or kombucha—by giving an intense, sharp aroma [30,31]. In this context, an interesting solution is the participation of LAB in reducing the level of acetic acid, which can result in an improved taste profile of the product. As shown by a study conducted by Wang et al. [32], the addition of LAB contributes to increased synthesis of alcohols and esters, thus softening the intense taste of vinegar.

Both malolactic fermentation and LAB activity have an additional impact on the functional properties of fermented foods. LAB can stimulate the production of bioactive compounds, such as peptides or secondary metabolites, that have beneficial health effects, especially in the context of intestinal functions [33,34]. Moreover, the presence of prebiotics and probiotics in these products also enhances their functional value, supporting potential health-promoting effects [33].

Despite the numerous benefits of reducing acetic acid and performing malolactic fermentation, it is necessary to maintain a balance between sensory and microbiological benefits. Excessive reduction of acetic acid can reduce its antimicrobial function, while malolactic fermentation is not always effective, especially in environments with high acidity and low pH, which can inhibit the growth of LAB [29].

### 2.2. Identification and Quantification of Polyphenolic Compounds

Analysis performed using ultra-performance liquid chromatography coupled with mass spectrometry (UPLC-Q-Tof-MS/MS, Waters, Manchester, UK) revealed the presence of 66 polyphenolic compounds from the group of phenolic acids, flavanols and procyanidins, purine alkaloids, theaflavins, flavonols and flavones in kombucha starter, 29 polyphenolic compounds from the group of polyphenolic acids and their derivatives, flavanols and procyanidins, flavonols and dihydrochalcones in apple juice, and 49 compounds from the group of polyphenolic acids and their derivatives, flavanols and procyanidins, flavonols, flavones and hydroquinones in pear juice (Table 2). The identification of polyphenolic compounds was made based on authentic standards and the available literature [35,36,37].

The identified compounds were typical for the raw materials used. In the kombucha starter, 21 compounds from the group of phenolic acids, mainly caffeic and gallic acid derivatives, were identified. The characteristic compound in this group was theogallin with [M-H]^-^ ions at *m/z* 343.0782 and theogallin dimer with [M-H]^-^ ions at *m/z* 687.1256. In the group of flavanols and procyanidins, 14 compounds were identified, mainly derivatives of (epi)-catechines and (epi)-gallocatechines. The presence of theasinensin-gallate with [M-H]^-^ ions at *m/z* 761.1357 was also demonstrated. A characteristic group of compounds for tea (*Camellia Sinensis*) are alkaloid purines. In this group, 2 compounds have been identified, such as theanine with [M-H]^-^ ions at *m/z* 173.0745 and theobromine with [M-H]^-^ ions at *m/z* 179.0652. Additionally, two theaflavins with [M-H]^-^ ions at *m/z* 563.0724 were identified. From the flavones group, apigenin-6,8-*C*-dipentoside with [M-H]^−^ ions at *m/z* 533.1366 was present in the tested kombucha.

In apple juice, 11 compounds from the phenolic acid group and their derivatives were identified. These were caffeic and coumaric acid and their derivatives. In the group of flavanols and procyanidins, 11 compounds were identified; those were monomers: (+)-catechin and (−)-epicatechin and dimers, trimers, and tetramers of procyanidins. The group of flavonols consisted of 6 quercetin glycosides. Compounds characteristic of apples were also identified, such as phloretin 2′-*O*-xyloglucoside ([M-H]^-^ ions at *m*/*z* 567.0808) and phloretin 2′-*O*-glucoside ([M-H]^-^ ions at *m*/*z* 435.0708) of the dihydrochalcone group.

In pear juice, 20 phenolic acids and their derivatives were identified, mainly caffeic acid, quinic acid, coumaric acid, syringic acid, and ferulic acid. In the group of flavanols and procyanidins, out of 16 identified compounds, 2 were monomers [(+)-catechin and (−)-epicatechin], 2 were double-charged penta- and heptameric procyanidins ([M-H]^−^ at *m*/*z* 720.6527 and *m*/*z* at 1008.2216, respectively), while the rest were procyanidin dimers, trimers, and tetramers. Among 12 flavonols, 3 kaempferol derivatives, 4 quercetin derivatives, and 5 isorhamnetin derivatives were observed. After the analysis, the presence of apigenin pentoside ([M-H]^−^ at *m*/*z* 401.1399) and arbutin ([M-H]^−^ ions at *m*/*z* 211.0855) was also observed.

The content of polyphenolic compounds in the tested kombucha starter was determined to be 117.16 mg/100 mL (Table 3). The dominant group of compounds were flavan-3-ols and proanthocyanidins, the content of which was 111.26 mg/100 mL of the beverage. The dominant compounds in this group were (−)-epigallocatechin and (−)-epicatechin gallate, which are compounds characteristic of tea [37]. The second largest group was phenolic acids and their derivatives (2.13 mg/100 mL), with gallic acid as the dominant compound (0.77 mg/100 mL). The third largest group was flavonols. In this group of compounds, kaempferol and quercetin derivatives were determined. The content of these compounds was at the level of 2.02 mg/100 mL. Compounds characteristic of tea, i.e., purine alkaloids and theaflavins, were present in the tested kombucha in small amounts—their contents constituted 1.08% and 0.4% of all polyphenolic compounds determined in kombucha, respectively.

The addition of 10% of the kombucha starter to apple juice (Table 4) and pear juice (Table 5) did not significantly affect the polyphenol composition of the juices in the initial stage of fermentation (day 0). The results obtained showed that there were no significant changes in the total phenolic content immediately after adding kombucha, but subtle modifications were observed in the composition of individual groups of compounds.

In the case of apple juice (Table 4), the total phenolic content decreased minimally from 1569.3 to 1562.3 mg/L. The most stable group was phenolic acids, whose sum increased slightly (from 415.4 to 431.4 mg/L), which may indicate the activation of enzymatic processes leading to the release of these compounds from bound forms. Among the compounds in this group, the increase in the content of 4-Caffeoylquinic acid is particularly noteworthy. In the case of flavanols and procyanidins, there was a slight decrease (from 1107.3 to 1085.9 mg/L), which may be the result of their transformation or partial degradation in the presence of microorganisms. Flavonols and dihydrochalcones showed a slight decrease in content, but these changes did not have a significant effect on the total content of polyphenols.

In pear juice (Table 5), high stability of the phenolic profile was also observed after the addition of kombucha. The total content of phenolic compounds decreased only slightly (from 515.0 to 492.9 mg/L). Phenolic acids remained practically unchanged (188.0 vs. 184.6 mg/L), showing only minimal changes in individual compounds. Flavanols and procyanidins decreased moderately (from 264.7 to 248.3 mg/L), which may indicate their enzymatic degradation or the beginning of conversion to other metabolites. Flavonols also showed a decreasing trend (from 17.3 to 15.1 mg/L), while the content of flavones and hydroquinones (including arbutin) did not change significantly.

The results obtained suggest that the addition of 10% of the kombucha starter does not lead to significant changes in the total phenolic content of the juices tested in the short term (up to 24 h). However, the observed minor differences in phenolic profiles may indicate the initial biotransformation processes induced by microorganisms present in the kombucha beverage. Kombucha itself is characterized by a high content of phenolic compounds, including epigallocatechin gallate (49.21 mg/100 mL) and epicatechin gallate (47.22 mg/100 mL). These compounds exhibit strong antioxidant properties and are easily metabolized by fermenting microorganisms, including yeast and acetic acid bacteria present in the kombucha culture. The tested juices did not show the presence of purine alkaloids and gallocatechins, which may be due to the small addition of starter or be due to the chromatographic conditions used; however, in both cases, it can be assumed that the high concentration of catechins and gallates present in green tea acted as a source of easily digestible phenolic substrates, which allowed microorganisms to focus their metabolism on these compounds while limiting the intensive degradation of fruit-derived components. In apple and pear juices, the profile of greatest changes in the phenolic compounds was visible only after 14 days of fermentation, suggesting that the presence of tea polyphenols (especially epigallocatechin gallate, catechin gallate, and gallic acid) modulates the course of fermentation, influencing both the rate of decomposition of juice polyphenols and the formation of secondary bioactive metabolites.

The fermentation process caused statistically significant changes in the content of phenolic compounds in both juices tested (Table 4 and Table 5). Detailed data on the change in the content of individual compounds during 14 days of fermentation are provided in Tables S1 and S2. A reduction in total polyphenol concentration in apple juice was observed, decreasing from 1562.3 mg/L before fermentation to 1263.6 mg/L after 14 days (Table 4). The primary components were flavonols and procyanidins, with their overall concentration declining from 1085.9 mg/L to 747.0 mg/L. The predominant procyanidin tetramer type A (343.00 mg/L) was detected, which, during fermentation, transformed into a trimer of the same type (178.6 mg/L), which subsequently reduced the tetramer concentration to 106.9 mg/L.

Fermentation influenced the polymer structure, resulting in a reduction of highly polymerized procyanidins and an increase in (epi)-catechin monomers. On the initial day of the procedure, the concentration of (+)-catechin was 37.3 mg/L, and (−)-epicatechin was 45.1 mg/L; after 14 days, these concentrations increased to 91.91 mg/L and 54.0 mg/L, respectively. This behavior is elucidated by the activities of microorganisms within the SCOBY consortium, which degrade polymers into monomeric forms [38].

The subsequent largest group consisted of phenolic acids, whose quantity grew as fermentation advanced. 4-caffeoylquinic acid was the predominant compound, with a concentration of 299.2 mg/L prior to fermentation and 295.4 mg/L after fermentation. Dihydrochalcones increased from 27.9 mg/L to 31.1 mg/L, with flavoretin glucoside (phloretin 2′-*O*-glucoside) being predominant, increasing from 23.9 mg/L to 27.4 mg/L. Flavonols, the least abundant group of chemicals examined, exhibited a reduction in concentration, particularly quercetin-3-O-rhamnoside (6.5 mg/L). This reduction may be due to glycoside hydrolysis and the use of sugar residues by bacteria [38].

The overall concentration of polyphenolic components in pear juice increased from 492.9 mg/L to 576.7 mg/L (Table 4). Before fermentation, flavonols and procyanidins were predominant, while after fermentation, phenolic acids prevailed. Their content increased from 184.6 mg/L to 239.2 mg/L, while the concentrations of flavonols and procyanidins decreased from 248.3 mg/L to 164.2 mg/L. Among the phenolic acids, 4-caffeoylquinic acid showed a substantial increase from 140.0 mg/L to 168.8 mg/L. The most significant reduction among flavonols and procyanidins occurred with procyanidin trimer type A, which decreased to 9.6 mg/L, while dimer type B emerged as the most prevalent at 44.1 mg/L after the end of the process.

A notable increase in hydroquinone concentration was recorded, escalating from 40.8 mg/L to 155.9 mg/L, with arbutin as the only chemical identified in this category. The second to last category by amount consisted of flavonols, whose concentration increased from 15.1 mg/L to 16.5 mg/L; the predominant molecule was acylated hexose isorhamnetin, which increased from 3.3 mg/L to 3.9 mg/L. The least abundant group consisted of flavones, with their concentration decreasing from 4.1 mg/L to 0.9 mg/L. Only apigenin pentoside was identified in this group.

SCOBY fermentation plays a key role in the modification of the chemical profile of kombucha, including a significant impact on the content of polyphenolic compounds. The microorganisms present in SCOBY, including acetic acid bacteria and various yeast species, carry out a series of biochemical transformations that not only increase the total polyphenol content but also lead to the formation of new phenolic compounds with potential biological properties. The fermentation process results in a significant increase in the level of total phenols and flavonoids. As shown in studies, the content of these compounds can increase up to 3.53 and 5.2 times, respectively, after fermentation with the participation of SCOBY [39]. Furthermore, the presence of tea leaf residues during fermentation also intensifies this effect—an increase in polyphenol concentration was observed up to 5.68 times compared to samples without their presence [40]. This phenomenon indicates the important role of the availability of plant substrates in the intensification of the extraction and transformation of phenolic compounds. During fermentation, new phenolic compounds are synthesized and released, such as chlorogenic, p-coumaric, or ferulic acid, which were not present in the unfermented infusion. The enzymatic activity of SCOBY microorganisms also leads to the hydrolysis of glycosidic bonds, resulting in the formation of free aglycones such as gallic acid or quercetin [41]. Such transformations increase both the bioavailability and the biological activity of these compounds. The fermentation process affected the ratio of marked groups of phenolic compounds. Furthermore, the fermentation carried out decomposes the complex polyphenols contained in the raw material into simpler, more bioavailable compounds in the final product [38,42]. This is related to the use of polyphenols by microorganisms as substrates during fermentation. Macromolecular polyphenols, such as proanthocyanidins and flavonoid glycosides, decompose into smaller phenolic compounds, such as aglycone, ellagic acid, and catechin [38]. In raw materials that contain a significant amount of proanthocyanidins (blueberries, strawberries), the content of these compounds decreases after fermentation. It is caused by the metabolism of anthocyanins to free phenolic acids by probiotic microorganisms [38]. Polyphenols also affect the growth of the microorganisms contained in the product; they cause the growth of probiotic bacteria and prevent the development of pathogenic microorganisms [42]. It is worth mentioning that the effectiveness of fermentation in increasing the content and diversity of polyphenols depends on many factors, including the microbiological composition of SCOBY and the parameters of the fermentation process. Differences in the strains of bacteria and yeast used, fermentation time, or type of substrate can lead to different effects in the context of the amount and type of synthesized phenolic compounds [43,44]. This variability highlights both the complexity of fermentation mechanisms and the potential for their optimization to maximize the health effects of fermented beverages.

### 2.3. Determination of Biological Activities

Kombucha beverages have several health benefits, including anti-inflammatory, antioxidant, anticancer, antidiabetic, and antibacterial characteristics [13]. The antibiotic activities of the tea fungus extract are attributed to medusin, a natural antibiotic generated by the fungus [11], as well as additional bioactive chemicals or metabolites formed during fermentation [45]. When ingested in moderation, it may have health-promoting properties. To investigate the effect of fermentation on the biological properties of fermented juices, antioxidant, antidiabetic, anti-inflammatory, and anti-aging properties were determined in apple and pear juices (Table 5).

To explore the influence of fermentation on the pro-health properties of fermented apple and pear juices, their biological activities were investigated (Table 6).

#### 2.3.1. Antioxidant Capacity

The analysis showed that the fermentation process had a negative effect on the antioxidant activity of apple juice (Table 6). The highest values were observed on the sixth day of the process. Up to the 6th day, the values increased and then decreased intensively. The value of the ABTS parameter decreased the most, by 622.61 μM Tx/100 mL. The DPPH test showed a decrease of 355.64 μM Tx/100 mL, while the smallest decrease was determined for the FRAP value of 285.33 μM Tx/100 mL.

In pear juice, fermentation had a positive effect on the properties tested. All parameters had a higher final value than the initial one, while the highest activity was determined on the 6th day of fermentation, after which the values changed and decreased. The largest increase was shown in the FRAP parameter by 561.23 μM Tx/100 mL, followed by ABTS by 181.21 μM Tx/100 mL and DPPH of 3.14 μM Tx/100 mL.

Based on these results, it can be concluded that pear juice proved to be a better raw material for SCOBY fermentation than apple juice. This juice created better conditions to increase the parameters being determined. Additionally, the observations carried out show that the optimal time to increase the activity of the fruit juices tested is 6 days.

The antioxidant activity of kombucha is one of the key parameters of its biological quality. Its intensity reaches maximum values at different times of fermentation, depending on the substrate used. For example, kombucha obtained from fruits—especially strawberries—showed high antioxidant activity, with the maximum phenol content recorded during fermentation, although the best moment to end the process was not clearly defined [16]. In turn, another study showed that in the case of green tea-prepared kombucha, the highest level of antioxidant activity (93.79%) was achieved on the seventh day of fermentation [21]. This indicates significant differences depending on the type of plant material used and potentially other factors, such as the environmental conditions of fermentation or the composition of the SCOBY microflora.

In the study by Pawluś and Kolniak-Ostek [10], it was found that the fermentation process had a negative effect on the values of the ABTS parameter. A decrease in the parameter value was observed in all kombucha variants tested. The value of the FRAP parameter decreased in two of the four variants tested (K1 and K2). On the other hand, in the settings (K3 and K4), in which a higher fermentation temperature (37 °C) was used, the activity tested increased. Furthermore, in variant (K4), in which the highest addition of sucrose (15%) was used, the highest increase was observed. This suggests that the increase in temperature and the larger amount of sugar had a positive effect on the value of the FRAP parameter. The results of the DPPH test also showed that after 16 days of fermentation, the parameter value decreased in all variants. After the DPPH test, it was found that in two variants (K3 and K4) of the kombuchas tested, the highest values were recorded before fermentation. In the first two variants (K1 and K2), the highest DPPH test values were observed on the 4th day of the process. It was found that the temperature of 20 °C, at which the fermentation was carried out, favors the maximum increase in the DPPH value in the initial 4 days of the process. Nurikasari et al. [21] also investigated the antioxidant activity of kombucha using the DPPH assay. This study showed an increase in activity during the fermentation process. The highest activity was observed on the 7th day of the process, and on the following days (9 and 11), the values were lower. This suggests that the optimal fermentation time to obtain the highest results is 7 days. The increase in activity occurred as a result of the metabolism of microorganisms present in the batch during fermentation. The decrease in activity is due to the acidic environment, which makes phenolic compounds more stable and has difficulty releasing a proton that can bind to DPPH, and for this reason, the activity tested decreased [21]. Polyphenols, including flavan-3-ols, isocoumarins, and their derivatives, flavonols, are natural compounds with a broad spectrum of biological activity, including anti-inflammatory, antioxidant, and anticancer properties. These compounds exhibit antioxidant properties, which were confirmed in DPPH radical scavenging tests. This mechanism may be related to the presence of hydroxyl groups in the ortho position, which allows the formation of resonance stable structures, which favors the neutralization of reactive oxygen species [46,47].

#### 2.3.2. Antidiabetic Activity

The fermentation process increased the antidiabetic activity in the analyzed fruit juices (Table 6). Apple juice showed a more significant increase compared to pear juice. An increase in α-amylase activity was observed in apple juice by 51.46% and in pear juice by 33.44%, and α-glucosidase in apple juice by 49.94% and in pear juice by 36.64%. This is due to the greater amount of sugars present in the fruit. Substances such as organic acids and microbial metabolites were formed, which influenced the increase in the activity tested. Additionally, the amount of sugar contained decreased due to fermentation, which influenced the antidiabetic properties. In a study conducted by Geraris Kartelias et al. [48], it was shown that Olympus Mountain tea (*Sideritis scardica*) sweetened with honey showed a strong inhibitory effect on α-amylase and α-glucosidase. Furthermore, this activity increased after the fermentation process. Phenolic substances, by inhibiting α-glucosidase and α-amylase, can have a significant effect on carbohydrate and lipid metabolism. It is possible to inhibit their actions through their ability to chelate, changes in structure, and possible limitations of biological activities. α-Amylase and α-glucosidase are important in the process of carbohydrate digestion, playing a key role in their metabolism. By reducing the action of these enzymes, the intensity of glucose absorption into the bloodstream decreases, resulting in a decrease in blood glucose levels [48,49].

Fruit juices and kombucha, as sources of bioactive compounds such as polyphenols and antioxidants, are gaining importance as potential elements supporting dietary therapy for diabetes. Fruits are widely considered to be metabolically beneficial, but the assessment of the impact of their processed forms—especially juices—remains ambiguous due to the high content of simple sugars. Although fruit juices contain numerous protective substances, their natural sugar content raises concerns about their impact on blood glucose levels. The polyphenols present in fruits have hypoglycemic effects, among others, by inhibiting enzymes that digest carbohydrates, improving insulin sensitivity, and limiting glucose absorption [50,51]. Resveratrol and anthocyanins, as selected members of this group, have been associated with reduced fasting glucose and HbA1c levels [52], although human studies are still inconsistent [53]. Similarly, antioxidants, by reducing oxidative stress, may improve glycemic control, and fruits such as berries and citrus have been associated with a reduced risk of type 2 diabetes [54,55]. However, meta-analyses indicate that 100% fruit juice consumption does not significantly affect fasting glucose and insulin levels [56,57]. The glycemic effect can vary depending on the type of juice; for example, berry juices may have a more beneficial effect [57]. However, due to the high sugar content, moderate consumption of juices is recommended, especially in people with carbohydrate metabolism disorders [58]. Kombucha, on the other hand, as a fermented tea drink, has promising antihyperglycemic properties. Due to the presence of polyphenols and organic acids, it can support glucose regulation and improve insulin sensitivity. The polyphenols contained in kombucha inhibit starch-digesting enzymes (α-amylase and α-glucosidase), limiting glucose absorption [50], and antioxidant compounds reduce oxidative stress [59,60]. In a pilot study, consumption of kombucha for four weeks reduced fasting glucose levels from 164 to 116 mg/dl [58], and animal experiments have shown a reduction of 56% in glycemia [61]. These benefits may also result from the effect of the drink on the gut microbiota: it increases the number of bacteria producing short-chain fatty acids (SCFAs), which support metabolic health and insulin sensitivity [62,63]. Despite promising reports, more well-designed clinical trials are needed for both fruit juices and kombucha. In the case of kombucha, in particular, differences resulting from fermentation methods and microbial composition, which can affect efficacy, should be taken into account. Only high-quality human studies will confirm the therapeutic potential of these products and establish the optimal principles of their use in the context of glycemic control in people with diabetes.

#### 2.3.3. Anticholergenic Activity

Both fermented juices showed anticholinergic activity, with the highest anti-aging activity observed on the 14th day of pear juice fermentation (Table 6). AChE (acetylcholinesterase) activity increased by 8.08% from 11.31% enzyme inhibition to 19.39% enzyme inhibition, while BuChE (butyrylcholinesterase) increased by 11.04% from 14.06% enzyme inhibition to 25.10% enzyme inhibition. In apple juice, AChE activity also increased by 7.80% from 10.76% enzyme inhibition to 18.56% enzyme inhibition and BuChE by 9.02% from 10.28% enzyme inhibition to 19.30% enzyme inhibition, but is lower than in the second variant. It can be concluded that SCOBY fermentation had a positive effect on the health properties of fruit juices. In the article by Pawluś and Kolniak-Ostek [10], an increase in the activity tested was found in each variant of kombucha. The highest inhibitory activity was shown by the fermented variant at an elevated temperature (37 °C) and with a smaller amount of sucrose (10%).

In the study by Geraris Kartelias et al. [49], it was shown that the addition of spices such as hibiscus calyx, rose petals, and ginger root to kombucha resulted in an increase in inhibitory anti-aging activity. This is related to the content of bioactive compounds in the added raw materials. The inhibitory effect of acetylcholinesterase and butyrylcholinesterase is used in the treatment of Alzheimer’s disease [49].

#### 2.3.4. Anti-Inflammatory Activity

In each variant, there was an increase in anti-inflammatory activity, the largest of which was observed in the case of the COX-2 enzyme in pear juice; the value increased from 36.68% to 61.48% (Table 6). On the other hand, the COX-1 value on the 14th day of fermentation was 81.73%. In apple juice, the highest activity was observed in COX-1, which was 93.91%; however, the COX-2 enzyme showed the lowest value among the tested samples, 42.48%. After the analysis, it can be concluded that the fermentation process will have a positive effect on the anti-inflammatory activity of both mixtures. In the study by Kolniak-Ostek et al. [3], it was found that the average ability of compounds found in pear extracts to inhibit the activity of the COX-1 enzyme was 50.53%, and the COX-2 enzyme was 58.15%. The extracts analyzed showed greater anti-inflammatory activity of the COX-2 enzyme than of COX-1 by approximately 15%. These enzymes are different from each other. COX-1 is a constitutive cyclooxygenase that determines the proper and correct functioning of internal organs because it influences the regulation of prostanoid synthesis. On the other hand, the activity of the induced COX-2 isoform increases in response to stress factors and inflammation. Compounds that inhibit the activity of this enzyme have anticancer potential and are mainly used as anti-inflammatory drugs [3]. Polyphenols are a broad group of plant compounds with significant pharmacological significance, many of which exhibit strong anti-inflammatory effects. This effect is achieved in multiple ways by modulating pro-inflammatory enzymes, inhibiting cytokine expression, and stabilizing the cellular structures involved in inflammatory responses. In studies by Ramanan et al. [64], isocoumarins—natural phenolic compounds—were shown to act as effective inhibitors of key enzymes involved in the inflammatory pathways of arachidonic acid metabolism, such as 5-lipoxygenase (5-LOX) and microsomal prostaglandin E2 synthase (mPGES1). In particular, compound 1c, a derivative of 3-arylisocoumarin, showed dual inhibitory effects on both enzymes (IC₅₀ 4.6 ± 0.26 µM for 5-LOX and 6.3 ± 0.13 µM for mPGES1, respectively). A decrease in the expression level of the COX-2 and mPGES1 genes was also observed, indicating the influence of these compounds at the transcriptional level. In turn, a study by Wahyanto and Agustini [65] presented the anti-inflammatory properties of flavonoids contained in a fermented drink, kombucha, enriched with Clitoria terna tea flower extract. Flavonoids, such as quercetin and its glycosides, showed the ability to stabilize human erythrocyte membranes (lysosomal model) and reduce their susceptibility to hemolysis, which was considered evidence of anti-inflammatory activity. The lowest IC₅₀ value was 140.22 ppm (kombucha with 2% added extract), indicating moderate but clear anti-inflammatory efficacy. The mechanism of action of flavonoids includes, among others, inhibition of enzymes, phospholipase A2 (PLA2), cyclooxygenase (COX), lipoxygenase (LOX), and nitric oxide synthase (NOS), which leads to a reduction in the production of inflammatory mediators such as PGE2, leukotrienes, and nitric oxide. Furthermore, flavonoids affect the activity of transcription factors (e.g., NF-κB) and regulate the expression of cytokines (including TNF-α, IL-6), resulting in the suppression of the inflammatory response. Similar biological properties are also suggested by Sudarshan [46], who presented the synthesis of glycosidated derivatives of isocoumarins—novel structures connecting an isocoumarin ring with a sugar unit. Although the main emphasis was placed on synthetic aspects, these structures, because of the presence of a phenolic and a glycosidic part, exhibit potential anti-inflammatory properties analogous to quercetin and its derivatives.

### 2.4. Chemometric Analysis

**Figure 1:**
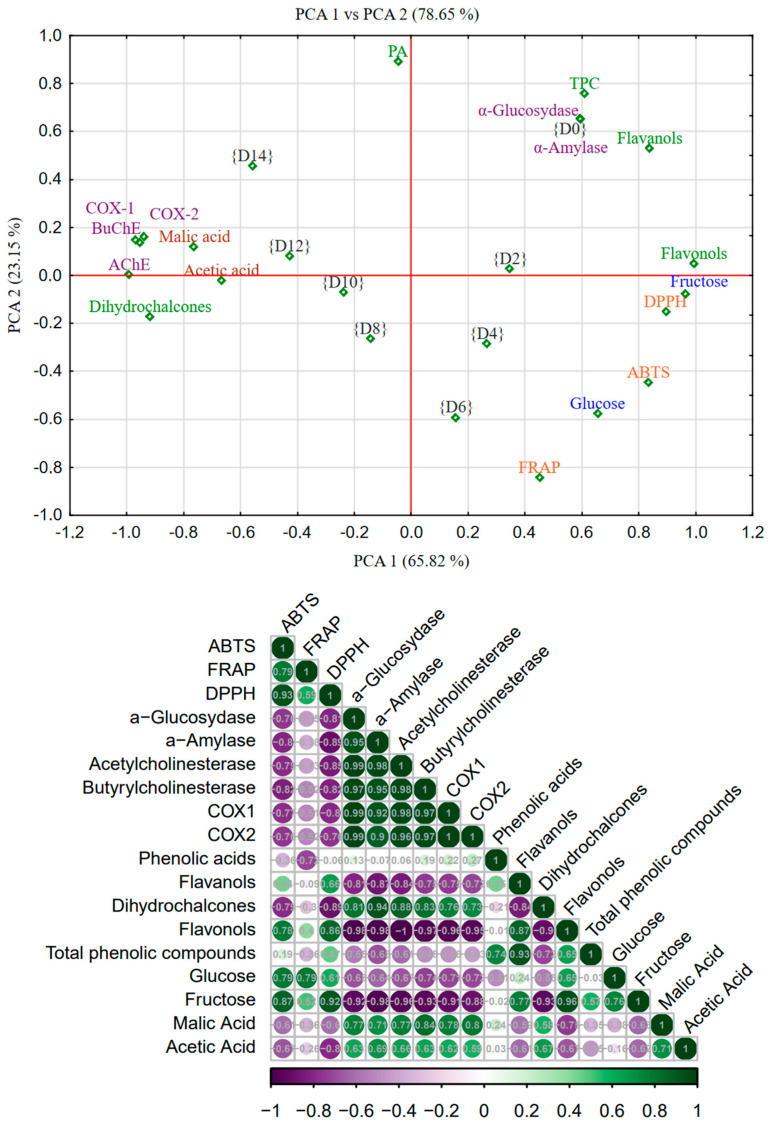
Principal component analysis, a score plot of the first two principal components of fermented apple juice. The PCA score plot depicts the relationship between the chemical composition of kombucha and its biological activity, the degree of correlation between the variables (individual phenolic groups, organic acids, reducing sugars, and biological activities), and specific principal components (PCs) in fermented apple juice. The normalized factor loadings plot assigns physical meaning to each primary component. The loading indicates the level of association between the two variables (particular phenolic groups, organic acids, reducing sugars, and biological activities) and specific principal components (PCs). Abbreviations: {D0}–{D14}—days of fermentation; TPC—total phenolic compounds; PA—phenolic acids.

On the basis of the presented correlation graph, significant relationships can be observed between the chemical compounds, enzymes, and antioxidant properties tested of SCOBY-fermented apple juices (Figure 1).

The conducted principal component analysis (PCA) clearly indicates the influence of the fermentation process on the chemical composition and bioactive properties of apple juice. In Figure 1, two groups of samples can be observed: samples from the initial days of fermentation {D0}-{D6}, characterized by a high content of simple sugars and polyphenolic compounds from the flavonols and flavanols group. The presence of these groups of polyphenol compounds is positively correlated with the antioxidant properties of the juice (ABTS, DPPH, and FRAP) and the antidiabetic activity. Additionally, a negative correlation can be observed between simple sugars and antidiabetic activity. On the other hand, a group of juices from the second half of fermentation {D8}-{D24} can be observed, in which there are high concentrations of organic acids and low concentrations of glucose and fructose (negative correlation). These samples are positively correlated with high health-promoting properties, such as anti-inflammatory activity (COX-1 and COX-2) and anticholergenic activity (AChE and BuChE).

The strongest positive correlations occur between antioxidant activity (ABTS, FRAP, DPPH) and the content of phenolic compounds, such as flavonols, flavonols, phenolic acids, or dihydrochalcones. The correlation between DPPH and flavanols is particularly clear (r = 0.66), suggesting that these compounds are largely responsible for antioxidant activity. On the other hand, we observed strong negative correlations between the content of simple sugars (glucose, fructose) and organic acids (especially malic acid) and enzymatic activity and phenolic compounds. For example, glucose is negatively correlated with phenolic acids and flavonols. This may suggest that a higher concentration of these compounds in fruits occurs in samples less rich in sugars and organic acids. Strong negative correlations can also be observed between the sugar content and the activity of enzymes such as acetylcholinesterase or α-glucosidase—for example, fructose correlates with acetylcholinesterase activity at the level of r = −0.95. This indicates a potential antagonism between sugar profiles and enzymatic activity.

In the correlation analysis, it is worth paying special attention to enzymes related to the inflammatory and nervous systems: COX-1, COX-2, acetylcholinesterase (AChE), and butyrylcholinesterase (BChE). COX-1 and COX-2 (cyclooxygenases) are enzymes responsible for the synthesis of prostaglandins, which play a key role in inflammatory processes. The presented correlation matrix shows strongly negative associations of these enzymes with the level of sugars. For example, COX-1 and COX-2 correlate with glucose at a level of about −0.70, while with fructose at a level of about −0.8 to −0.9, respectively, which suggests that higher concentrations of these compounds may significantly inhibit the activity of inflammatory enzymes. A strong positive correlation was observed between COX-1 and COX-2 and the content of acids—malic (r = 0.8) and acetic (r = 0.6). The presented correlation matrix shows strongly negative associations of these enzymes with the level of phenolic compounds, particularly flavonols and dihydrochalcones. For example, COX-2 is correlated with flavonols at a level of approximately −0.72, suggesting that higher concentrations of these compounds may significantly inhibit the activity of inflammatory enzymes. This is particularly important in the context of the anti-inflammatory properties of apples: high concentrations of phenolic compounds can potentially act as natural COX inhibitors. In turn, acetylcholinesterase (AChE) and butyrylcholinesterase (BChE) are enzymes that degrade the neurotransmitter acetylcholine. Their excessive activity is associated with neurodegeneration, e.g., in Alzheimer’s disease. Correlations indicate that the activity of AChE and BChE also shows negative associations with the content of phenolic compounds, especially flavonols, flavonols, and dihydrochalcones (r = −0.29 to −0.72).

In summary, both inflammatory enzymes (COX-1, COX-2) and cholinesterases (AChE, BChE) show significant, mainly negative correlations with bioactive phenolic compounds present in apples. This may indicate the potential anti-inflammatory and neuroprotective effects of these fruits, especially in varieties with high flavonoid content.

**Figure 2:**
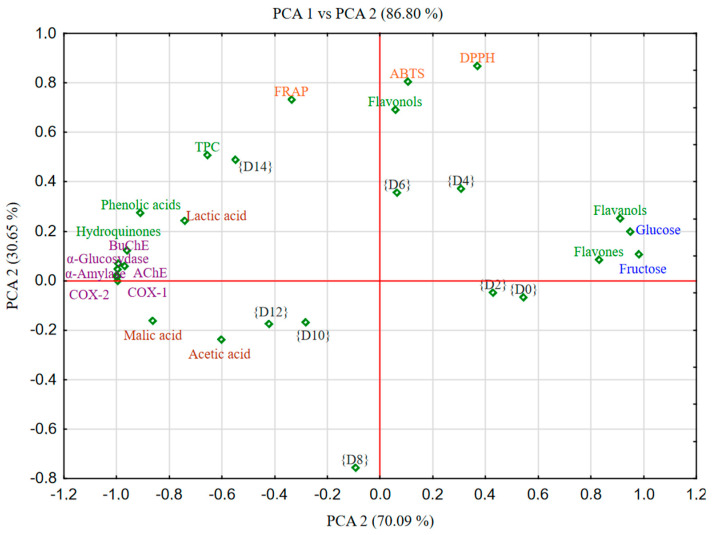

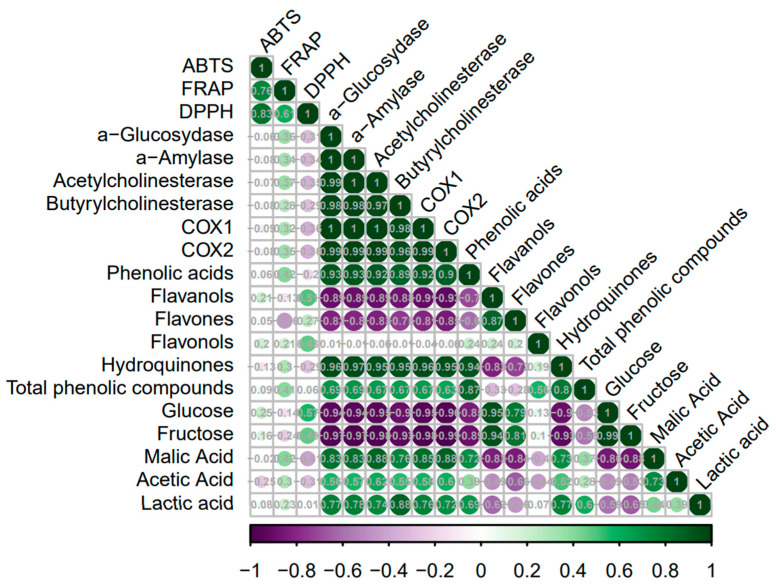
The degree of connection between the variables (particular phenolic groups, organic acids, reducing sugars, and biological activities) and specific principle components (PCs) in fermented pear juice. The degree of connection between the variables (particular phenolic groups, organic acids, reducing sugars, and biological activities) and specific principle components (PCs) in fermented pear juice. The normalized factor loadings plot assigns physical meaning to each primary component. The loading indicates the level of association between the two variables (particular phenolic groups, organic acids, reducing sugars, and biological activities) and the specific principal components (PCs). Abbreviations: {D0}–{D14}—days of fermentation; TPC—total phenolic compounds.

Based on the correlation matrix presented for fermented pear juice, clear dependence patterns can be observed between the parameters studied: antioxidant activity, enzymatic activity, phenolic content, and basic chemical components such as sugars and organic acids (Figure 2).

In the case of pear juices, similar relationships can be observed as in apple juices. Samples from the initial days of fermentation {D0}–{D6} are positively correlated with a high content of simple sugars and polyphenolic compounds from the flavanols and flavones group. This group of compounds, together with flavonols, has a direct effect on the value of the antioxidant capacity of fermented pear juices. In samples from the second half of the fermentation process {D8}–{D14}, a high content of organic acids, total phenolic compounds, in particular hydroquinones and phenolic acids, can be observed. These compounds are directly and positively correlated with the biological activity of pear juices.

Positive correlations occur between antioxidant parameters (ABTS, FRAP, DPPH) and the content of phenolic compounds, especially flavonols, flavones, hydroquinones, and the total content of phenolic compounds. For example, DPPH shows a positive correlation with flavanols (r = 0.51) and flavonols (r = 0.48), indicating that these compounds are largely responsible for the antioxidant activity of pears. In the case of FRAP, a positive correlation can be observed with phenolic acids (r = 0.42), hydroquinones (r = 0.30), and total phenolic compounds (r = 0.41). In the case of ABTS, a positive correlation can be observed with flavanols (r = 0.21) and flavonols (r = 0.20). Cyclooxygenases (COX-1 and COX-2), responsible for the formation of inflammatory mediators, show clearly positive correlations with the content of phenolic compounds. These correlations reach even r = 0.98 in the case of hydroquinones, which indicates a very strong effect of these compounds on the potential inhibition of pro-inflammatory enzyme activity. Similarly to apples, AChE and BChE in pears also show weak or moderate negative correlations with some phenolic compounds but are much weaker associated than COXs. This may suggest a smaller effect of phenolic compounds on these enzymes in pears than in apples.

Simple sugars (glucose, fructose) generally show negative correlations with the biological activity of fermented pear juices, which means that fruits with higher sugar content are characterized by lower bioactivity.

In summary, pears with a higher content of flavonoids, especially flavonols and hydroquinones, show stronger antioxidant activity and potential anti-inflammatory effects. Fruits richer in sugars have a weaker bioactive profile. This indicates the possibility of selecting pear varieties for their health-promoting properties through the analysis of the chemical composition.

## 3. Materials and Methods

### 3.1. Raw Material and Sample Processing

The research material consisted of freshly pressed juice from ‘Konferencja’ pears and freshly pressed juice from ‘Ligol’ apples, purchased in a retail store in 2024. A 10% (*v*/*v*) of a starter consisting of mature kombucha (created after 10 days of fermentation in *Camellia sinensis* infusion and distilled water, with the addition of sucrose in the amount of 60 g/1000 mL) was added to each juice. The juices were fermented in a dark room for 14 days at 22 °C. The samples for analysis were taken before fermentation began (day 0) and then every other day. Three yeast strains (*Saccharomyces cerevisiae*, *Saccharomyces ludwigii*, and *Schizosaccharomyces pombe*), two acetic acid bacterial strains (*Acetobacter pasteurans* and *Acetobacter aceti*), and two lactic acid bacterial strains (*Lactobacillus acidophilus* and *Lactobacillus fermentum*) comprised the SCOBY culture. Microorganisms were grown in the following particular growth media: Sabuardo agar with chloramphenicol for yeast, MRS agar for lactic acid bacteria, and Acetobacter agar for Acetobacter.

The pH, total soluble solids, reducing sugars and organic acids, identification of phenolic compounds, as well as antioxidant capacity, anti-inflammatory, antidiabetic, and anticholinergic properties were determined for each sample.

### 3.2. Physicochemical Analyses 

The total soluble solids (TSS) content was determined using an Atago PR-101 digital refractometer (Atago Co. Ltd., Tokyo, Japan) and expressed as °Brix. pH was determined with an automatic DL-21 titerator (Mettler-Toledo, Schwerzenbach, Switzerland).

### 3.3. Determination of Sugars and Organic Acids

Sugar determination was carried out with a Merck-Hitachi L-7455 liquid chromatograph (Merck KgaA, Darmstadt, Germany), while organic acid determination was carried out with a Dionex liquid chromatograph (Sunnyvale, CA, USA), according to the methods described by Chandran et al. 2025 [37]. Determinations were made in triplicate (*n* = 3) and are presented in grams per 100 mL of beverage.

### 3.4. UPLC-PDA-Q/Tof-MS Analysis of Polyphenolic Compounds

For the determination of phenolic compounds, the method described by Chandran et al. 2025 [37] was used. An ACQUITY UPLC system equipped with a PDA detector and a G2 Q-Tof mass detector (Waters, Manchester, UK) with an ESI source operating in negative and positive mode was used. Polyphenols were separated using a UPLC BEH C18 column (1.7 μm, 2.1 × 100 mm, Waters) at 30 °C for 15 min. The mobile phase included 0.1% formic acid (*v*/*v*) (solvent A) and 100% acetonitrile (solvent B), with a linear gradient sequence and constant flow rates of 0.42 mL/min. The runs were measured at the following wavelengths: 254 nm for flavonols, 280 nm for flavan-3-ols, hydroquinones, and dihydrochalcones, 320 nm for phenolic acids, 340 nm for flavones, and 360 nm for flavonol glycosides. The PDA spectra were obtained in 2 nm steps across the wavelength range of 200–600 nm. Retention times and spectra were compared with authentic standards. The analysis was performed using full scan, data-dependent MS scanning from *m*/*z* 100 to 2500. At a concentration of 500 pg/mL, leucine enkephalin (*m*/*z* 554.2615 Da) served as the reference substance. The optimal MS settings were 2500 V capillary voltage, 30 V cone voltage, 100 °C source temperature, 300 °C desolvation temperature, and 300 L/h nitrogen flow rate.

All determinations were made in triplicate (*n* = 3) and are presented in micrograms per 100 mL of beverage.

### 3.5. Determination of Biological Activities: Antioxidant, Anti-Inflammatory, Antidiabetic, and Anticholinergic

All activities were determined according to the methods described by Chandran et al. 2025 [37] using a Synergy H1 microplate reader (BioTek, Winooski, VT, USA). Antioxidant activity was determined in triplicate using ABTS, FRAP, and DPPH assay methods and expressed as millimol of Trolox equivalent per 100 mL of drink. Anti-inflammatory activity was measured as a measure of inhibition of cyclooxygenase (COX-1, COX-2) in triplicate (*n* = 3) and presented as % inhibition. Antidiabetic activity was measured by inhibition tests of alpha-amylase and alpha-glucosidase. Measurements were made in triplicate (*n* = 3) and presented as % inhibition. Anticholinergic activity was presented as the ability to inhibit the activity of the acetylcholinesterase (AChE) and butyrylcholinesterase (BuChE) enzymes. The determinations were made in triplicate (*n* = 3) and expressed as % inhibition.

### 3.6. Statistical Analysis

Means (*n* = 3) were compared using Duncan’s test and one-way ANOVA (*p* < 0.05). All statistical analyses, including the principal component analysis (PCA), were performed with Statistica 13.3 (StatSoft, Kraków, Poland). The Factoextra R 1.0.7 package was used to perform chemometric analysis.

## 4. Conclusions

The fermentation process using the SCOBY culture significantly affected the chemical composition and biological properties of the apple and pear juices. Despite the reduction in the total content of polyphenolic compounds in apple juice, structural transformations of these compounds were observed, especially the reduction of highly polymerized procyanidins to monomeric forms, such as (+)-catechin and (−)-epicatechin. In the case of pear juice, the total content of polyphenols increased, which indicates the enzymatic activity of microorganisms that leads to the hydrolysis of glycosidic bonds and the release of free aglycones with higher bioavailability and biological activity. These transformations had a direct effect on the antioxidant properties of the samples tested. In apple juice, an initial increase in antioxidant activity was observed, reaching a maximum on the sixth day of fermentation, followed by a decrease, which may be related to further degradation of phenols in the acidic fermentation environment. In the case of pear juice, antioxidant activity increased more steadily, reaching final values higher than before fermentation. This may be due to the presence of phenolic acids such as chlorogenic and p-coumaric acid, the content of which increased during fermentation. An increase in anti-inflammatory, antidiabetic, and anticholinesterase activity was also observed in apple and pear juices, which confirms the beneficial effect of fermentation on the functional properties of the products. This effect may be related not only to the presence of transformed phenolic compounds but also to the synergistic effect of secondary metabolites, such as organic acids and yeast autolysis products.

The research results confirm that fermentation using the SCOBY culture is an effective method to increase the biological value of fruit juices by transforming polyphenolic compounds into more active forms and improving their antioxidant properties. This process can be used in the production of functional foods supporting the prevention of lifestyle diseases.

## Figures and Tables

**Table 1:** pH value, total soluble solids (°Brix), reducing sugars (g/100 mL), and organic acids (g/100 mL) in apple and pear fruit juices during fermentation.

				Reducing Sugars		Organic Acids		
		pH	Total Soluble Solids	Fructose	Glucose	Malic Acid	Acetic Acid	Lactic Acid
Fresh apple juice		3.22 ± 0.01 ^de^	12.70 ± 0.00 ^a^	1.19 ± 0.01 ^a^	0.36 ± 0.01 ^a^	0.71 ± 0.01 ^b^	0.10 ± 0.01 ^e^	—
Apple juice	Day 0	3.19 ± 0.00 ^e^	11.75 ± 0.07 ^bc^	0.92 ± 0.02 ^c^	0.26 ± 0.02 ^bc^	0.57 ± 0.02 ^c^	0.07 ± 0.00 ^ef^	—
	Day 2	3.19 ± 0.06 ^e^	12.00 ± 0.14 ^c^	0.90 ± 0.01 ^c^	0.29 ± 0.01 ^b^	0.60 ± 0.03 ^c^	0.08 ± 0.01 ^e^	—
	Day 4	3.14 ± 0.00 ^f^	11.40 ± 0.00 ^c^	0.73 ± 0.03 ^d^	0.28 ± 0.02 ^b^	0.58 ± 0.03 ^c^	0.05 ± 0.00 ^f^	—
	Day 6	3.14 ± 0.00 ^f^	11.30 ± 0.00 ^cd^	0.71 ± 0.02 ^d^	0.36 ± 0.03 ^a^	0.69 ± 0.02 ^b^	0.09 ± 0.01 ^e^	—
	Day 8	3.14 ± 0.00 ^f^	7.50 ± 0.00 ^f^	0.47 ± 0.02 ^f^	0.35 ± 0.02 ^a^	0.75 ± 0.03 ^b^	0.13 ± 0.00 ^d^	—
	Day 10	3.14 ± 0.05 ^f^	6.60 ± 0.00 ^g^	0.16 ± 0.01 ^h^	0.14 ± 0.01 ^d^	0.55 ± 0.00^c^	0.09 ± 0.01 ^e^	0.01 ± 0.00 ^a^
	Day 12	3.10 ± 0.00 ^g^	7.20 ± 0.00 ^f^	0.17 ± 0.01 ^h^	0.15 ± 0.00 ^d^	0.88 ± 0.02 ^a^	0.11 ± 0.01 ^de^	0.03 ± 0.00 ^a^
	Day 14	3.10 ± 0.00 ^g^	5.70 ± 0.00 ^i^	0.11 ± 0.00 ^i^	0.09 ± 0.00 ^e^	0.88 ± 0.02 ^a^	0.11 ± 0.01 ^de^	0.03 ± 0.00 ^a^
Fresh pear juice		4.40 ± 0.03 ^a^	12.35 ± 0.10 ^b^	0.96 ± 0.01 ^ab^	0.18 ± 0.01 ^cd^	0.15 ± 0.01 ^e^	0.33 ± 0.01 ^b^	—
Pear juice	Day 0	4.12 ± 0.00 ^b^	11.60 ± 0.00 ^c^	1.02 ± 0.03 ^b^	0.23 ± 0.02 ^c^	0.18 ± 0.00 ^e^	0.35 ± 0.02 ^b^	—
	Day 2	4.07 ± 0.03 ^b^	12.30 ± 0.00 ^b^	0.97 ± 0.02 ^b^	0.24 ± 0.02 ^c^	0.19 ± 0.01 ^e^	0.37 ± 0.02 ^ab^	—
	Day 4	3.71 ± 0.00 ^bc^	11.00 ± 0.00 ^d^	0.68 ± 0.01 ^d^	0.15 ± 0.01 ^d^	0.21 ± 0.02 ^e^	0.29 ± 0.00 ^c^	—
	Day 6	3.58 ± 0.00 ^c^	8.95 ± 0.07 ^e^	0.59 ± 0.02 ^e^	0.15 ± 0.01 ^d^	0.32 ± 0.01 ^d^	0.40 ± 0.02 ^a^	—
	Day 8	3.51 ± 0.00 ^c^	6.70 ± 0.00 ^g^	0.27 ± 0.01 ^g^	0.03 ± 0.00 ^f^	0.31 ± 0.02 ^d^	0.38 ± 0.01 ^a^	—
	Day 10	3.48 ± 0.00 ^c^	6.10 ± 0.00 ^h^	0.13 ± 0.00 ^h^	---	0.31 ± 0.01 ^d^	0.39 ± 0.02 ^a^	—
	Day 12	3.35 ± 0.00 ^d^	6.30 ± 0.00 ^gh^	0.06 ± 0.01 ^i^	---	0.32 ± 0.00 ^d^	0.40 ± 0.01 ^a^	0.03 ± 0.00 ^a^
	Day 14	3.32 ± 0.00 ^d^	5.60 ± 0.00 ^i^	0.02 ± 0.00 ^j^	---	0.30 ± 0.02 ^d^	0.39 ± 0.00 ^a^	0.03 ± 0.00 ^a^

Means of three independent analyses ± standard deviation; Values in the same columns followed by different letters (a–j) are significantly different at *p* < 0.05 according to Duncan’s test.

**Table 2:** Retention times and characteristic ions of phenolic compounds of kombucha starter, apple, and pear juices.

Rt (min)	[M–H]^−^ (*m*/*z*) ^1^	MS/MS Fragments (*m*/*z*) ^1^	Tentative Identification	Kombucha Starter	Apple Juice	Pear Juice
*Phenolic acids*						
0.66	341.0574	191.0167	Caffeoylhexose	-	√	-
0.77	517.0757	387.5185/307.4093/163.9868	Coumaric acid derivative	-	√	-
0.88	341.1230	191.8541/173.0211	Caffeoylhexose	√	-	-
0.94	341.0571	174.9891	Caffeoylhexose	-	√	-
1.10	335.1482	179.0955	Caffeoylshikimic acid	√	-	-
1.28	341.0566	190.9808	Caffeoylhexose	√	√	√
1.42	341.0550	173.0659	Coffeoylhexose	-	-	√
1.44	609.1277	291.0152/173.1445	Gallocatechin dimer	√	-	-
1.46	331.0330	168.9945	Galloylhexose	√	-	-
1.59	169.0122	125.0368/107.0263/97.1233	Gallic acid ^2^	√	-	-
1.67	191.0185	173.9566	Quinic acid ^2^	-	-	√
1.69	331.0330	168.9945	Galloylhexose	√	-	-
1.78	343.0782	191.0030	Theogallin ^2^	√	-	-
1.84	687.1256	343.0778/191.1543	Theogallin dimer	√	-	-
1.85	341.0603		Caffeoylhexose	-	√	-
2.06	341.0465	173.0659	Coffeoylhexose	-	-	√
2.43	341.1326	191.1225	Coffeoylhexose	√	-	-
2.47	341.1152	191.0152/163.1143	Coffeoylhexose	√	-	-
2.59	365.0780	229.0397	Caffeoyl *N*-tryptophan	-	-	√
2.67	359.0391	197.0391	Syryngic acid galactoside	-	-	√
2.78	163.0436		*p*-Coumaric acid ^2^	-	-	√
2.90	353.0316	191.0148	3-Caffeoylquinic acid ^2^	-	-	√
2.91	635.0922	483.1077/465.1228/313.0702	Trigalloylhexose	√	-	-
3.03	353.0136	191.0553. 179.2167	*cis*-3-Caffeoylquinic acid	-	-	√
3.14	325.0641	307.1234	*p*-Coumaric acid 4-*O*-glucoside	√	-	-
3.16	337.0658	173.0507	*cis*-4-*p*-Coumaroylquinic acid	-	-	√
3.34	483.0769	331.0214/168.2301	1,6-Digalloyl glucose	√	-	-
3.46	353.0822	191.0544	5-Caffeoylquinic acid ^2^	-	-	√
3.53	355.1933	193.0247	Ferulic acid hexoside	-	-	√
3.75	359.0921	197.0046. 153.9228	Syryngic acid glucoside	-	-	√
3.81	385.0677	223.1583	Sinapic acid hexoside	-	-	√
3.95	337.0536	191.0531, 163.0162	trans-5-*p*-Coumaroyloquinic acid	√	√	-
3.96	483.0803	331.0214/169.9105	1,6-Digalloyl glucose	√	-	-
4.12	353.0346	191.0164	4-Caffeoylquinic acid ^2^	-	√	√
4.23	353.0339	191.0165	*cis*-4-Caffeoylquinic acid	-	√	-
4.32	511.0567	365.0225/265.2115/63.0394	Caffeoyl-N-tryptophanrhamnoside	√	-	-
4.33	295.0978	179.0328	Caffeoyl-l-malic acid	-	-	√
4.46	325.0833	191.0125. 163.0313	*p*-Coumaroylhexose	-	-	√
4.71	511.0813	341.0654/179.0573	Cafeoyl galloyl hexoside	√	-	-
4.78	337.0924	191.0236	5-*p*-Coumaroyloquinic acid	√	-	-
5.07	353.0282	191.0159	1-Caffeoylquinic acid	-	√	√
5.23	337.0408	191.0079	*cis*-5-*p*-Coumaroyloquinic acid	√	√	-
5.54	337.1267	173.0612	3-*p*-Coumaroylquinic acid	√	-	√
7.90	515.1266	353.0355. 191.0623	di-*O*-Caffeoylquinic acid	-	-	√
*Flavanols and procyanidins*						
2.19	609.2322	305.0269	Gallocatechin dimer	√	-	-
2.61	305.0691	219.4306/191.0652	(+)-Gallocatechin ^2^	√	-	-
3.21	577.1326	289.1136	B-type Procyanidin dimer	-	-	√
3.27	557.1322	289.1022	B-type Procyanidin dimer	-	-	√
3.59	761.1357	609.1226/591.2341	Theasinensin-gallate	√	-	-
3.65	305.1302	125.1401	(−)-Epigallocatechin ^2^	√	-	-
3.75	577.0678	289.0214	B-type Procyanidin dimer	-	√	-
3.93	1153.2258	865.1234. 577.1993. 287.0715	B-type Procyanidin tetramer	-	-	√
4.00	577.1326	289.0043	B-type Procyanidin dimer	-	-	√
4.11	915.1637	457.1220/169.5023	(−)-Epigallocatechin gallate ^2^	√	-	-
4.23	577.1277	289.1456	B-type Procyanidin dimer	-	-	√
4.26	1153.2217	865.1937. 577.1322. 389.1134	B-type Procyanidin tetramer	-	-	√
4.30	865.1090	575.0361; 289.0272	B-type Procyanidin trimer	√	√	-
4.45	1154.1523	865.1246; 575.0493; 289.0390	B-type Procyanidin tetramer	-	√	√
4.46	577.1509	289.1345	B-type Procyanidin dimer	√	-	-
4.56	1153.2258	865.1234. 577.1993. 287.0715	B-type Procyanidin tetramer	-	-	√
4.72	865.1023	577.0698; 289.0229	B-type Procyanidin trimer	-	√	-
4.73	577.0820	289.0180	B-type procyanidin dimer	-	-	√
4.88	1153.1634	865.1252; 287.0016	A-type Procyanidin tetramer	-	√	-
4.90	865.2100	577.1254. 289.1344	B-type Procyanidin trimer	-	-	√
4.95	1153.1844	577.0530; 289.0229	B-type Procyanidin tetramer	-	√	-
5.13	289.0236	245.0487	(+)-Catechin ^2^	√	√	√
5.15	457.0715	305.1266/275.0324	Gallocatechin gallate	√	-	-
5.28	863.1920	577.1320. 289.0818	A-type Procyanidin trimer	-	-	√
5.45	720.6527	577.1333. 389.0677	Double-charged pentameric procyanidin	-	-	√
5.48	865.1158	287.0067	A-type Procyanidin trimer	-	√	-
5.76	1008.2216	864.1896. 287.0190	Double-charged heptameric procyanidin	-	-	√
5.78	1153.1429	865.1124; 577.0516; 289.0265	B-type Procyanidin tetramer	-	√	-
5.90	557.1646	289.0289	B-type Procyanidin dimer	-	√	-
6.00	865.1998	577.1558. 287.1135	B-type Procyanidin trimer	-	-	√
6.38	577.1345	289.0246	B-type Procyanidin dimer	√	-	-
6.70	441.0845	289.0263	(+)-Epicatechin gallate	√	-	-
6.81	289.0241	245.0475	(−)-Epicatechin ^2^	√	√	√
6.96	597.1932	291.1346	A-type procyanidin dimer ^2^	√	-	-
7.57	723.2398	289.1250	Catechin dimer + deoxyhexose	√	-	-
*Purine Alkaloids*						
0.80	173.0745	156.0213/82.1153	Theanine ^2^	√	-	-
2.55	179.0652	136.1205	Theobromine ^2^	√	-	-
*Theaflavins*						
5.74	563.0724	443.0628/315.0266	Theaflavin unknown isomer	√	-	-
5.79	563.0724	443.0628/315.0266	Theaflavin unknown isomer	√	-	-
*Flavonols*						
5.20	593.1326	285.0312	Kaempferol 3-*O*-*p*-coumaroylhexoside	√	-	-
6.01	771.0100	301.0104	Quercetin 3-*O*-glucosylrutinoside	√	-	-
6.14	593.1500	285.1526	Kaempferol 3-*O*-*p*-coumaroylhexoside	√	-	-
6.21	771.2050	301.0523	Quercetin 3-*O*-glucosylrutinoside	√	-	-
6.30	755.2150	301.0098	Quercetin 3-*O*-dirhamnosylhexoside	√	-	-
6.36	739.1573	593.1212; 285.7339	Kaempferol hexoside-dideoxyhexoside	-	-	√
6.45	755.2154	285.1326	Kaempferol 3-*O*-glucosylrutinoside	√	-	-
6.50	609.0786	301.0698	Quercetin 3-*O*-rutinoside	-	-	√
6.59	463.0210	301.0354	Quercetin 3-*O*-galactoside ^2^	√	-	√
6.71	463.0331	301.0355	Quercetin 3-*O*-glucoside ^2^	√	√	√
6.85	623.1700	315.0551	Isorhamnetin 3-*O*-rhamnosyl-hexoside	-	-	√
6.86	755.2154	285.0314	Kaempferol 3-*O*-glucosylrutinoside	√	-	-
6.98	463.0329	301.1265	Quercetin-3-*O*-hexoside	-	√	-
7.14	433.0100	301.0014	Quercetin-3-*O*-xyloside	-	√	-
7.20	593.1542	285.0321	Kaempferol 3-*O*-*p*-coumaroylhexoside	√	-	-
7.35	505.0515	463.0900. 301.0354	Quercetin-acylated-hexoside	-	-	√
7.47	623.0863	315.0551	Isorhamnetin 3-*O*-rhamnosyl-hexoside	-	-	√
7.48	433.0256	301.9845	Quercetin-3-*O*-arabinoside	-	√	-
7.61	477.1220	315.0551	Isorhamnetin 3-*O*-galactoside	-	-	√
7.66	447.0241	301.1111	Quercetin-3-*O*-rhamnoside ^2^	-	√	-
7.74	447.0816	285.0312	Kaempferol 3-*O*-glucoside ^2^	√	-	√
8.11	433.1159	301.1111	Quercetin-3-*O*-pentoside	-	√	-
8.16	477.1022	315.0551	Isorhamnetin 3-*O*-glucoside	-	-	√
8.4	489.1042	285.0312	Kaempferol 3-*O*-6-acetylated-glucoside	-	-	√
8.68	1079.3120	539.1263/301.1121	Quercetin 3-*O*-acylglycoside	√	-	-
8.89	1079.2259	539.1259/301.1120	Quercetin 3-*O*-acylglycoside	√	-	-
8.96	917.2359	609.0244/301.0112	Quercetin 3-*O*-*p*-coumaroyl-glucosyl-rhamnosyl-galactoside	√	-	-
9.02	1049.2788	301.0133	Quercetin 3-*O*-acylglycoside	√	-	-
9.16	1063.2851	285.0321	Kaempferol 3-*O*-acylglycoside	√	-	-
9.25	885.2170	301.0211	Quercetin 3-*O*-*p*-coumaroyl-dirhamnosyl-hexoside	√	-	-
9.28	519.1166	315.0457	Isorhamnetin-acylated-hexoside	-	-	√
9.32	1063.2983	285.1332	Kaempferol 3-*O*-acylglycoside	√	-	-
9.40	1049.2754	301.0133	Quercetin 3-*O*-acylglycoside	√	-	-
9.44	1049.2854	301.0133	Quercetin 3-*O*-acylglycoside	√	-	-
9.53	901.2348	301.1254	Quercetin 3-*O*-*p*-coumaroyl-glucosyl-rutinoside	√	-	-
9.64	1063.2983	301.0211	Quercetin 3-*O*-acylglycoside	√	-	-
9.77	1033.2848	285.1532	Kaempferol 3-*O*-acylglycoside	√	-	-
9.85	917.2359	609.0244/301.0112	Quercetin 3-*O*-*p*-coumaroyl-glucosyl-rhamnosyl-galactoside	√	-	-
9.93	1047.2903	285.1322	Kaempferol 3-*O*-*p*-coumaroyl-rhamnosyl-dihexoside	√	-	-
*Flavones*						
5.86	401.1399	269.1803	Apigenin pentoside	-	-	√
7.00	533.1366	433.1183/291.1219	Apigenin-6,8-*C*-dipentoside	√	-	-
*Hydroquinones*						
1.85	211.0855		Arbutin ^2^	-	-	√
*Dihydrochalcones*						
7.83	567.0808	273.8322	Phloretin 2′-*O*-xyloglucoside ^2^	-	√	-
8.58	435.0708	273.0303	Phloretin 2′-*O*-glucoside ^2^	-	√	-

^1^ Experimental data. ^2^ Identified using corresponding authentic standards.

**Table 3:** The content of phenolic compounds (mg/100 mL) in kombucha starter (average ± standard deviation; *n* = 3).

Kombucha Starter	
Compound	mg/100 mL
*Phenolic acids*	
Caffeoylhexose	0.01 ± 0.00
Caffeoylshikimic acid	0.04 ± 0.00
Caffeoylhexose	0.01 ± 0.00
Gallocatechin dimer	0.01 ± 0.00
Galloylhexose	0.02 ± 0.00
Gallic acid ^1^	0.77 ± 0.01
Galloylhexose	0.08 ± 0.00
Theogallin	0.28 ± 0.01
Theogallin dimer	0.09 ± 0.00
Caffeoylhexose	0.01 ± 0.00
Caffeoylhexose	0.01 ± 0.00
Trigalloylhexose	0.20 ± 0.01
*p*-Coumaric acid 4-*O*-glucoside	0.11 ± 0.01
1,6-Digalloyl glucose	0.03 ± 0.00
trans-5-*p*-Coumaroylquinic acid	0.18 ± 0.01
1,6-Digalloyl glucose	0.02 ± 0.00
Caffeoyl-*N*-tryptophanrhamnoside	0.05 ± 0.00
Cafeoyl galloyl hexoside	0.04 ± 0.00
5-*p*-Coumaroylquinic acid	0.12 ± 0.01
*cis*-5-*p*-Coumaroylquinic acid	0.04 ± 0.00
3-*p*-Coumaroylquinic acid	0.03 ± 0.00
Sum	2.13 ± 0.05
*Flavan-3-ols and Proanthocyanidins*	
Gallocatechin dimer	0.02 ± 0.00
(+)-Gallocatechin	0.02 ± 0.00
Theasinensin-gallate	0.11 ± 0.01
(−)-Epigallocatechin	5.66 ± 0.02
(−)-Epigallocatechin gallate	49.21 ± 0.16
B-type procyanidin trimer	0.25 ± 0.01
B-type procyanidin dimer	0.14 ± 0.00
(+)-Catechin ^1^	0.17 ± 0.01
Gallocatechin gallate	2.50 ± 0.01
B-type procyanidin dimer	0.11 ± 0.00
(+)-Epicatechin gallate	47.22 ± 0.09
(−)-Epicatechin ^1^	5.79 ± 0.01
A-type procyanidin dimer	0.01 ± 0.00
Catechin dimer + deoxyhexose	0.04 ± 0.00
Sum	111.26 ± 0.59
*Purine Alkaloids*	
Theanine ^1^	0.12 ± 0.01
Theobromine ^1^	1.15 ± 0.01
Sum	1.27 ± 0.05
*Theaflavins*	
Theaflavin unknown isomer	0.22 ± 0.00
Theaflavin unknown isomer	0.25 ± 0.01
Sum	0.47 ± 0.06
*Flavonols*	
Kaempferol 3-*O*-*p*-coumaroylhexoside	0.04 ± 0.00
Quercetin 3-*O*-glucosylrutinoside	0.09 ± 0.00
Kaempferol 3-*O*-*p*-coumaroylhexoside	0.08 ± 0.00
Quercetin 3-*O*-glucosylrutinoside	0.26 ± 0.01
Quercetin 3-*O*-dirhamnosylhexoside	0.02 ± 0.00
Kaempferol 3-*O*-glucosylrutinoside	0.38 ± 0.00
Quercetin 3-*O*-galactoside ^1^	0.04 ± 0.00
Quercetin 3-*O*-glucoside ^1^	0.03 ± 0.00
Kaempferol 3-*O*-glucosylrutinoside	0.49 ± 0.02
Kaempferol 3-*O*-*p*-coumaroylhexoside	0.11 ± 0.00
Kaempferol 3-*O*-glucoside ^1^	0.03 ± 0.00
Quercetin 3-*O*-acylglycoside	0.08 ± 0.00
Quercetin 3-*O*-acylglycoside	0.03 ± 0.00
Quercetin 3-*O*-p-coumaroyl-glucosyl-rhamnosyl-galactoside	0.01 ± 0.00
Quercetin 3-*O*-acylglycoside	0.05 ± 0.00
Kaempferol 3-*O*-acylglycoside	0.11 ± 0.01
Quercetin 3-*O*-*p*-coumaroyl-dirhamnosyl-hexoside	0.01 ± 0.00
Kaempferol 3-*O*-acylglycoside	0.02 ± 0.00
Quercetin 3-*O*-acylglycoside	0.02 ± 0.00
Quercetin 3-*O*-acylglycoside	0.03 ± 0.00
Quercetin 3-*O*-p-coumaroyl-glucosyl-rutinoside	0.02 ± 0.00
Quercetin 3-*O*-acylglycoside	0.01 ± 0.00
Kaempferol 3-*O*-acylglycoside	0.01 ± 0.00
Quercetin 3-*O*-*p*-coumaroyl-glucosy-lrhamnosyl-galactoside	0.02 ± 0.00
Kaempferol 3-*O*-*p*-coumaroyl-rhamnosyl-dihexoside	0.02 ± 0.00
Sum	2.02 ± 0.05
*Flavones*	
Apigenin-6,8-*C*-dipentoside	0.01 ± 0.00
TOTAL	117.16 ± 1.26

^1^ Identified using corresponding authentic standards.

**Table 4:** The content of phenolic compounds (mg/100 mL) in apple juice before and after fermentation (average ± standard deviation; *n* = 3).

	Apple Juice		
Compound	Fresh Juice	Day 0	Day 14
*Phenolic acids*			
Caffeoylhexose	3.2 ± 0.1 ^a^	3.2 ± 0.1 ^a^	1.5 ± 0.1 ^c^
Coumaric acid derivative	36.5 ± 0.8 ^a^	36.4 ± 0.5 ^a^	31.2 ± 1.0 ^b^
Caffeoylhexose	6.7 ± 0.3 ^ab^	6.6 ± 0.2 ^ab^	6.3 ± 0.1 ^b^
Caffeoylhexose	4.6 ± 0.1 ^d^	4.4 ± 0.1 ^d^	22.1 ± 0.5 ^a^
Caffeoylhexose	37.0 ± 1.2 ^e^	37.6 ± 0.8 ^e^	71.8 ± 1.2 ^a^
trans-5-*p*-Coumaroyloquinic acid	4.9 ± 0.0 ^a^	4.9 ± 0.1 ^a^	3.6 ± 0.0 ^c^
4-Caffeoylquinic acid	281.6 ± 3.6 ^a^	299.2 ± 2.6 ^a^	295.4 ± 3.2 ^a^
cis-4-Caffeoylquinic acid	10.1 ± 0.5 ^a^	9.3 ± 0.2 ^ab^	8.8 ± 0.3 ^b^
1-Caffeoylquinic acid	9.5 ± 0.3 ^b^	9.3 ± 0.2 ^b^	8.8 ± 0.1 ^c^
cis-5-*p*-Coumaroyloquinic acid	21.3 ± 0.7 ^a^	20.6 ± 0.5 ^b^	20.8 ± 0.4 ^ab^
Sum	415.4 ± 6.9 ^b^	431.4 ± 8.5 ^b^	470.2 ± 4.8 ^a^
*Flavanols and procyanidins*			
B-type Procyanidin dimer	47.5 ± 2.3 ^a^	45.8 ± 0.6 ^a^	30.5 ± 0.3 ^b^
B-type Procyanidin trimer	55.1 ± 1.5 ^a^	52.4 ± 0.3 ^a^	41.6 ± 0.2 ^b^
B-type Procyanidin tetramer	45.2 ± 0.9 ^a^	44.7 ± 0.2 ^a^	36.9 ± 0.3 ^c^
B-type Procyanidin trimer	123.2 ± 2.2 ^a^	118.5 ± 0.5 ^a^	93.6 ± 0.6 ^b^
A-type Procyanidin tetramer	345.9 ± 2.6 ^a^	343.00 ± 2.5 ^a^	106.9 ± 1.5 ^h^
B-type Procyanidin tetramer	33.6 ± 0.3 ^b^	31.4 ± 0.2 ^c^	36.1 ± 0.2 ^a^
(+)-Catechin	37.9 ± 0.5 ^f^	37.3 ± 0.5 ^f^	91.1 ± 0.8 ^a^
A-type Procyanidin trimer	280.9 ± 1.5 ^a^	279.5 ± 1.0 ^a^	178.6 ± 1.1 ^d^
B-type Procyanidin tetramer	42.6 ± 0.6 ^a^	41.7 ± 0.3 ^a^	32.9 ± 0.2 ^c^
B-type Procyanidin dimer	46.9 ± 0.3 ^b^	46.5 ± 0.3 ^b^	44.9 ± 0.3 ^b^
(−)-Epicatechin	48.5 ± 0.5 ^b^	45.1 ± 0.4 ^c^	54.0 ± 0.3 ^a^
Sum	1107.3 ± 10.2 ^a^	1085.9 ± 9.8 ^b^	747.0 ± 8.7 ^e^
*Flavonols*			
Quercetin-3-*O*-galactoside	1.6 ± 0.1 ^a^	1.4 ± 0.1 ^a^	0.9 ± 0.0 ^b^
Quercetin-3-*O*-glucoside	2.5 ± 0.0 ^a^	2.4 ± 0.1 ^a^	2.0 ± 0.1 ^b^
Quercetin-3-*O*-xyloside	1.2 ± 0.1 ^a^	0.9 ± 0.0 ^a^	0.7 ± 0.0 ^b^
Quercetin-3-*O*-arabinoside	2.1 ± 0.1 ^a^	2.0 ± 0.1 ^a^	1.5 ± 0.2 ^c^
Quercetin-3-*O*-rhamnoside	6.5 ± 0.2 ^a^	6.5 ± 0.2 ^a^	6.5 ± 0.2 ^a^
Quercetin-3-*O*-xyloside	4.0 ± 0.1 ^a^	3.9 ± 0.1 ^a^	3.7 ± 0.1 ^b^
Sum	17.9 ± 0.5 ^a^	17.1 ± 0.9^b^	15.3 ± 0.6 ^e^
*Dihydrochalcones*			
Phloretin 2′-*O*-xyloglucoside	4.1 ± 0.1 ^a^	3.9 ± 0.1 ^a^	3.8 ± 0.2 ^a^
Phloretin 2′-*O*-glucoside	24.6 ± 0.4 ^d^	23.9 ± 0.5 ^d^	27.4 ± 0.7 ^b^
Sum	28.7 ± 0.7 ^c^	27.9 ± 0.5 ^d^	31.1 ± 0.8 ^b^
TOTAL	1569.3 ± 9.9 ^a^	1562.3 ± 10.1 ^a^	1263.6 ± 11.0 ^c^

Means of three separate analyses ± standard deviation. Duncan’s test reveals significant differences (*p* < 0.05) between values in the same rows with different letters (a–f,h).

**Table 5:** The content of phenolic compounds (mg/100 mL) in pear juice before and after fermentation (average ± standard deviation; *n* = 3).

	Pear Juice		
Compound	Fresh Juice	Day 0	Day 14
*Phenolic acids*			
Coffeoylhexose	5.7 ± 0.1 ^c^	5.7 ± 0.1 ^c^	9.2 ± 0.3 ^a^
Coffeoylhexose	5.4 ± 0.1 ^e^	5.3 ± 0.0 ^e^	10.5 ± 0.2 ^a^
Quinic acid	3.0 ± 0.2 ^e^	3.0 ± 0.1 ^e^	7.3 ± 0.2 ^a^
Coffeoylhexose	2.3 ± 0.1 ^d^	2.2 ± 0.0 ^d^	4.0 ± 0.1 ^a^
Caffeoyl N-tryptophan	7.9 ± 0.3 ^b^	7.8 ± 0.4 ^b^	9.5 ± 0.3 ^a^
Syryngic acid galactoside	0.5 ± 0.0 ^d^	0.5 ± 0.0 ^d^	1.6 ± 0.1 ^a^
*p*-Coumaric acid	0.2 ± 0.0 ^c^	0.2 ± 0.0 ^c^	1.6 ± 0.2 ^a^
3-Caffeoylquinic acid	0.9 ± 0.0 ^c^	0.8 ± 0.0 ^d^	2.8 ± 0.1 ^a^
cis-3-Caffeoylquinic acid	1.3 ± 0.1 ^b^	1.2 ± 0.0 ^b^	2.8 ± 0.2 ^a^
cis-4-*p*-Coumaroylquinic acid	0.4 ± 0.0 ^cd^	0.3 ± 0.0 ^d^	1.7 ± 0.1 ^a^
5-Caffeoylquinic acid	0.3 ± 0.0 ^cd^	0.3 ± 0.0 ^cd^	0.7 ± 0.0 ^b^
Ferulic acid hexoside	1.3 ± 0.1 ^a^	1.2 ± 0.0 ^a^	0.2 ± 0.0 ^c^
Syryngic acid glucoside	0.9 ± 0.0 ^cd^	0.9 ± 0.0 ^cd^	2.1 ± 0.2 ^a^
Sinapic acid hexoside	0.9 ± 0.1 ^d^	0.8 ± 0.0 ^d^	3.1 ± 0.2 ^a^
4-Caffeoylquinic acid	142.3 ± 3.2 ^cd^	140.0 ± 5.2 ^d^	168.8 ± 4.9 ^a^
Caffeoyl-l-malic acid	8.4 ± 0.3 ^a^	8.3 ± 0.5 ^a^	3.6 ± 0.2 ^c^
p-Coumaroylhexose	1.8 ± 0.2 ^a^	1.8 ± 0.2 ^a^	0.0 ± 0.0 ^d^
1-Caffeoylquinic acid	3.0 ± 0.1 ^e^	2.9 ± 0.1 ^e^	7.2 ± 0.2 ^a^
3-*p*-Coumaroylquinic acid	0.4 ± 0.0 ^c^	0.4 ± 0.1 ^c^	1.1 ± 0.1 ^a^
di-*O*-Caffeoylquinic acid	1.1 ± 0.1 ^b^	1.0 ± 0.0 ^b^	1.5 ± 0.1 ^a^
Sum	188.0 ± 4.8 ^d^	184.6 ± 5.6 ^d^	239.2 ± 3.2 ^a^
*Flavanols and procyanidins*			
B-type Procyanidin dimer	15.5 ± 1.0 ^a^	15.2 ± 1.2 ^a^	6.3 ± 0.5 ^d^
B-type Procyanidin dimer	13.4 ± 0.6 ^a^	13.3 ± 0.4 ^a^	10.6 ± 0.4 ^c^
B-type Procyanidin tetramer	11.9 ± 0.3 ^a^	11.7 ± 0.2 ^a^	4.8 ± 0.0 ^e^
B-type Procyanidin dimer	6.9 ± 0.3 ^a^	6.8 ± 0.3 ^a^	6.5 ± 0.2 ^a^
B-type Procyanidin dimer	42.3 ± 1.9 ^ab^	40.6 ± 2.8 ^b^	36.8 ± 0.3 ^c^
B-type Procyanidin tetramer	26.1 ± 1.0 ^a^	24.0 ± 1.1 ^b^	0.0 ± 0.0 ^d^
B-type Procyanidin tetramer	10.9 ± 0.5 ^a^	10.1 ± 0.5 ^a^	10.1 ± 0.3 ^a^
B-type Procyanidin tetramer	12.5 ± 0.4 ^a^	11.0 ± 0.9 ^b^	5.0 ± 0.1 ^f^
B-type procyanidin dimer	11.9 ± 0.5 ^f^	10.4 ± 0.2^f^	44.1 ± 0.3 ^a^
B-type Procyanidin trimer	6.6 ± 0.2 ^a^	6.4 ± 0.2 ^a^	5.4 ± 0.2 ^b^
(+)-Catechin	10.5 ± 0.2 ^e^	9.9 ± 0.7 ^e^	17.9 ± 0.2 ^a^
A-type Procyanidin trimer	84.0 ± 1.9 ^a^	81.1 ± 2.7 ^a^	9.6 ± 0.1 ^g^
Double-charged pentameric procyanidin	1.9 ± 0.1 ^a^	1.4 ± 0.3 ^b^	0.6 ± 0.0 ^c^
Double-charged heptameric procyanidin	4.6 ± 0.2 ^ab^	3.8 ± 0.1 ^b^	1.9 ± 0.1 ^d^
B-type Procyanidin trimer	3.2 ± 0.1 ^b^	2.7 ± 0.1 ^c^	2.1 ± 0.1 ^d^
(−)-Epicatechin	2.5 ± 0.1 ^a^	0.0 ± 0.0 ^d^	2.5 ± 0.1 ^a^
Sum	264.7 ± 5.3 ^a^	248.3 ± 4.8 ^b^	164.2 ± 1.8 ^e^
*Flavonols*			
Kaempferol hexoside-dideoxyhexoside	0.6 ± 0.0 ^a^	0.5 ± 0.0 ^a^	0.3 ± 0.0 ^b^
Quercetin 3-*O*-rutinoside	0.8 ± 0.0 ^cd^	0.7 ± 0.0 ^d^	2.0 ± 0.1 ^a^
Quercetin 3-*O*-galactoside	1.3 ± 0.1 ^a^	1.1 ± 0.1 ^a^	0.4 ± 0.0 ^d^
Quercetin 3-*O*-glucoside	0.6 ± 0.0 ^a^	0.4 ± 0.0 ^a^	0.3 ± 0.0 ^b^
Isorhamnetin 3-*O*-rhamnosyl-hexoside	1.5 ± 0.1 ^b^	1.3 ± 0.1 ^c^	2.9 ± 0.2 ^a^
Quercetin-acylated-hexoside	2.1 ± 0.1 ^a^	1.9 ± 0.1 ^b^	2.4 ± 0.2 ^a^
Isorhamnetin 3-*O*-rhamnosyl-hexoside	2.1 ± 0.0 ^a^	1.8 ± 0.1 ^b^	2.1 ± 0.1 ^a^
Isorhamnetin 3-*O*-galactoside	1.5 ± 0.0 ^a^	1.5 ± 0.1 ^a^	0.3 ± 0.0 ^c^
Kaempferol 3-*O*-glucoside	1.8 ± 0.1 ^b^	1.8 ± 0.0 ^b^	0.4 ± 0.0 ^e^
Isorhamnetin 3-*O*-glucoside	0.3 ± 0.0 ^b^	0.1 ± 0.0 ^a^	0.0 ± 0.0 ^c^
Kaempferol 3-*O*-6-acetylated-glucoside	1.1 ± 0.1 ^b^	0.9 ± 0.0 ^bc^	1.5 ± 0.1 ^a^
Isorhamnetin-acylated-hexoside	3.6 ± 0.1 ^b^	3.3 ± 0.1 ^c^	3.9 ± 0.1 ^a^
Sum	17.3 ± 0.9 ^a^	15.1 ± 0.5 ^c^	16.5 ± 0.2 ^b^
*Flavones*			
Apigenin pentoside	4.1 ± 0.1 ^a^	4.1 ± 0.2 ^a^	0.9 ± 0.0 ^e^
*Hydroquinones*			
Arbutin	40.9 ± 1.8 ^e^	40.8 ± 2.8 ^e^	155.9 ± 2.9 ^a^
TOTAL	515.0 ± 5.2 ^b^	492.9 ± 8.9 ^b^	576.7 ± 10.5 ^a^

Means of three separate analyses ± standard deviation. Duncan’s test reveals significant differences (*p* < 0.05) between values in the same rows with different letters (a–g).

**Table 6:** Antioxidant (ABTS, FRAP, DPPH) [μM Tx/100 mL], anti-inflammatory (COX1, COX2), antidiabetic (α-amylase, α-glucosidase) and anti-aging (acetylcholinesterase, butyrylcholinesterase) activities [% of Inhibition] of apple and pear juice during fermentation.

	Days	DPPH	ABTS	FRAP	α-Glucosidase	α-Amylase	AChE	BuChE	COX1	COX2
		μM Tx/100 mL			[% of Inhibition]					
Fresh apple juice		590.99 ± 0.5 ^b^	1295.81 ± 1.2 ^d^	1097.24 ± 0.9 ^l^	10.87 ± 0.6 ^c^	5.69 ± 0.2 ^c^	10.70 ± 0.3 ^d^	10.27 ± 0.0 ^d^	88.60 ± 0.5 ^b^	36.12 ± 0.1 ^c^
Apple juice	Day 0	596.27 ± 0.4 ^b^	1297.7 ± 0.6 ^d^	1275.83 ± 0.5 ^h^	10.88 ± 0.5 ^c^	5.69 ± 0.1 ^c^	10.76 ± 0.1 ^d^	10.28 ± 0.1 ^d^	88.63 ± 0.7 ^b^	36.15 ± 0.2 ^c^
	Day 2	596.07 ± 0.2 ^b^	1351.18 ± 0.4 ^c^	1595.24 ± 0.8 ^c^						
	Day 4	604.33 ± 0.0 ^ab^	1530.51 ± 0.7 ^b^	1633.42 ± 0.4 ^b^						
	Day 6	613.18 ± 0.6 ^a^	1646.28 ± 0.1 ^a^	2138.81 ± 0.2 ^a^						
	Day 8	238.27 ± 0.7 ^d^	978.70 ± 0.2 ^g^	1510.67 ± 0.1 ^d^						
	Day 10	245.04 ± 0.2 ^c^	823.29 ± 0.9 ^i^	1388.73 ± 0.4 ^f^						
	Day 12	246.53 ± 0.8 ^c^	669.13 ± 1.0 ^j^	1300.47 ± 0.1 ^g^						
	Day 14	240.63 ± 0.1 ^bd^	675.09 ± 0.8 ^j^	990.5 ± 0.9 ^m^	60.82 ± 0.8 ^a^	57.15 ± 0.6 ^a^	18.56 ± 0.2 ^b^	19.30 ± 0.4 ^b^	93.91 ± 1.0 ^a^	42.48 ± 0.2 ^b^
Fresh pear juice		211.11 ± 1.4 ^f^	598.03 ± 0.8 ^k^	836.13 ± 1.5 ^p^	5.60 ± 0.3 ^d^	2.31 ± 0.3 ^d^	11.30 ± 0.1 ^c^	13.90 ± 0.4 ^c^	75.36 ± 0.2 ^d^	36.41 ± 0.2 ^c^
Pear juice	Day 0	215.86 ± 0.7 ^f^	811.96 ± 0.3 ^ii^	873.90 ± 1.0 ^o^	5.66 ± 0.1 ^d^	2.38 ± 0.2 ^d^	11.31 ± 0.2 ^c^	14.06 ± 0.2 ^c^	75.58 ± 0.6 ^d^	36.68 ± 0.3 ^c^
	Day 2	222.54 ± 0.5 ^e^	824.54 ± 0.1 ^i^	1129.27 ± 0.9 ^k^						
	Day 4	224.59 ± 0.4 ^e^	1184.45 ± 0.7 ^f^	1205.22 ± 1.1 ^i^						
	Day 6	226.87 ± 0.6 ^e^	1264.98 ± 0.9 ^e^	1642.86 ± 0.9 ^b^						
	Day 8	197.46 ± 0.9 ^h^	677.31 ± 0.2 ^j^	941.23 ± 0.6 ^n^						
	Day 10	206.11 ± 0.1 ^g^	677.73 ± 0.4 ^j^	1151.44 ± 0.8 ^j^						
	Day 12	212.40 ± 0.9 ^f^	896.90 ± 0.6 ^h^	1126.80 ± 0.7 ^k^						
	Day 14	219.00 ± 0.6 ^ef^	993.17 ± 0.9 ^g^	1435.13 ± 0.6 ^e^	42.30 ± 0.3 ^b^	35.82 ± 0.5 ^b^	19.39 ± 0.3 ^a^	25.10 ± 0.4 ^a^	81.73 ± 0.8 ^c^	61.48 ± 0.5 ^a^

Means of three independent analyses, with standard deviations. Duncan’s test reveals significant differences (*p* < 0.05) between values in the same columns denoted by different letters (a–p). Abbreviations: ABTS—2,2′-Azino-bis(3-ethylbenzothiazoline-6-sulfonic acid); DPPH—2,2-Diphenyl-1-(2,4,6-trinitrophenyl)hydrazyl; FRAP—ferric reducing antioxidant power assay; AChE—Acetylcholinesterase; BuChE—Butyrylcholinesterase.

## Data Availability

Data will be made available on request.

## Supplementary Materials

The following supporting information can be downloaded at: https://www.mdpi.com/article/10.3390/molecules30091940/s1, Table S1: The content of phenolic compounds (mg/100 mL) in apple juice during fermentation (average ± standard deviation; *n* = 3); Table S2: The content of phenolic compounds (mg/100 mL) in pear juice during fermentation (average ± standard deviation; *n* = 3); Table S3: The content [%] of individual polyphenolic compounds in pear juice in relation to their corresponding polyphenol groups during fermentation; Table S4: The content [%] of individual polyphenolic compounds in pear juice in relation to total phenolic content during fermentation; Table S5: The content [%] of individual polyphenolic compounds in apple juice in relation to their corresponding polyphenol groups during fermentation; Table S6: The content [%] of individual polyphenolic compounds in apple juice in relation to total phenolic content during fermentation; Table S7: The rate of the biotransformation reaction [mg/h] of polyphenolic compounds in pear juice during fermentation; Table S8: The rate of the biotransformation reaction [mg/h] of polyphenolic compounds in apple juice during fermentation.

## Abbreviations

The following abbreviations are used in this manuscript:

ABTS	2,2′-Azino-bis(3-ethylbenzothiazoline-6-sulfonic acid)
DPPH	2,2-Diphenyl-1-(2,4,6-trinitrophenyl)hydrazyl
FRAP	Ferric reducing antioxidant power assay
AChE	Acetylcholinesterase
BuChE	Butyrylcholinesterase
LAB	Lactic Acid Bacteria
AAB	Acetic Acid Bacteria

## References

[1] Nowak D., Gośliński M., Kłębukowska L. (2022). Antioxidant and Antimicrobial Properties of Selected Fruit Juices. Plant Foods Hum. Nutr..

[2] Lewko K. (2017). Leczenie Dobrą Dietą.

[3] Kolniak-Ostek J., Kłopotowska D., Rutkowski K.P., Skorupińska A., Kruczyńska D.E. (2020). Bioactive Compounds and Health-Promoting Properties of Pear (*Pyrus communis* L.) Fruits. Molecules.

[4] Zhang X., Liao X., Wang Y., Rao L., Zhao L. (2024). Health effects of fruit juices and beverages with varying degrees of processing. Food Sci. Hum. Wellness.

[5] Pabich M., Materska M. (2019). Biological Effect of Soy Isoflavones in the Prevention of Civilization Diseases. Nutrients.

[6] Kitajewska W., Szeląg W., Kopański Z., Maslyak Z., Sklyarov I. (2014). Choroby cywilizacyjne i ich prewencja. J. Clin. Healthc..

[7] Chwała W., Jankowicz-Szymańska A. (2024). Man in Health and Disease. Prevention and Management of Civilizational Diseases: International Scientific Conference, May 24, 2024, Tarnów, Poland. Health Promot. Phys. Act..

[8] Yao L., Zhang J., Lu J., Chen D., Song S., Wang H., Sun M., Feng T. (2023). Revealing the influence of microbiota on the flavor of kombucha during natural fermentation process by metagenomic and GC-MS analysis. Food Res. Int..

[9] Marsh A.J., O’Sullivan O., Hill C., Ross R.P., Cotter P.D. (2014). Sequence-based analysis of the bacterial and fungal compositions of multiple kombucha (tea fungus) samples. Food Microbiol..

[10] Pawluś P., Kolniak-Ostek J. (2024). Innovative Analogs of Unpasteurized Kombucha Beverages: Comparative Analysis of Mint/Nettle Kombuchas, Considering Their Health-Promoting Effect, Polyphenolic Compounds and Chemical Composition. Int. J. Mol. Sci..

[11] Nieumywakin I. (2018). Grzyb Herbaciany: Naturalny Uzdrowiciel.

[12] Kitwetcharoen H., Phung L.T., Klanrit P., Thanonkeo S., Tippayawat P., Yamada M., Thanonkeo P. (2023). Kombucha Healthy Drink—Recent Advances in Production, Chemical Composition and Health Benefits. Fermentation.

[13] de Oliveira P.V., da Silva Júnior A.H., de Oliveira C.R.S., Assumpção C.F., Ogeda C.H. (2023). Kombucha benefits, risks and regulatory frameworks: A review. Food Chem. Adv..

[14] Sanwal N., Gupta A., Bareen M.A., Sharma N., Sahu J.K. (2023). Kombucha fermentation: Recent trends in process dynamics, functional bioactivities, toxicity management, and potential applications. Food Chem. Adv..

[15] de Miranda J.F., Ruiz L.F., Silva C.B., Uekane T.M., Silva K.A., Gonzalez A.G.M., Fernandes F.F., Lima A.R. (2022). Kombucha: A review of substrates, regulations, composition, and biological properties. J. Food Sci..

[16] Morales D., Gutiérrez-Pensado R., Bravo F.I., Muguerza B. (2023). Novel kombucha beverages with antioxidant activity based on fruits as alternative substrates. LWT-Food Sci. Technol..

[17] Chong A.Q., Chin N.L., Talib R.A., Basha R.K. (2024). Modelling pH Dynamics, SCOBY Biomass Formation, and Acetic Acid Production of Kombucha Fermentation Using Black, Green, and Oolong Teas. Processes.

[18] Fonteles T.V., dos Santos A.Y.S., Linhares M.D.F.D., Miguel T.B.A.R., Miguel E.d.C., Rodrigues S. (2024). Metabolic responses of kombucha consortium fermentation upon ultrasound-processing. Food Chem. Adv..

[19] Tomar O. (2023). Determination of some quality properties and antimicrobial activities of kombucha tea prepared with different berries. Turk. J. Agric. For..

[20] Jakubczyk K., Gutowska I., Antoniewicz J., Janda K. (2021). Evaluation of Fluoride and Selected Chemical Parameters in Kombucha Derived from White, Green, Black and Red Tea. Biol. Trace Elem. Res..

[21] Nurikasari M., Puspitasari Y., Siwi R.P.Y. (2017). Characterization and analysis kombucha tea antioxidant activity based on long fermentation as a beverage functional. J. Glob. Res. Public Health.

[22] Zubaidah E., Yurista S., Rahmadani N.R. (2018). Characteristic of physical, chemical, and microbiological kombucha from various varieties of apples. IOP Conf. Ser. Earth Environ. Sci..

[23] Jakubczyk K., Kałduńska J., Kochman J., Janda K. (2020). Chemical Profile and Antioxidant Activity of the Kombucha Beverage Derived from White, Green, Black and Red Tea. Antioxidants.

[24] de Oliveira P.M., Santos L.P., Coelho L.F., Neto P.M.A., Sass D.C., Contiero J. (2021). Production of L (+) Lactic Acid by *Lactobacillus casei* Ke11: Fed Batch Fermentation Strategies. Fermentation.

[25] Sheeladevi A., Ramanathan N. (2011). Lactic Acid Production Using Lactic Acid Bacteria under Optimized Conditions. Int. J. Pharm. Biol. Arch..

[26] Costa S., Summa D., Semeraro B., Zappaterra F., Rugiero I., Tamburini E. (2020). Fermentation as a Strategy for Bio-Transforming Waste into Resources: Lactic Acid Production from Agri-Food Residues. Fermentation.

[27] Oyewole O.A., Yakubu J.G., Kalu J., Abdulfatah M.T., Abioye O.P., Adeniyi O.D., Egwim E.C. (2023). Microbial conversion of agro-wastes for lactic acid production. Sci. Afr..

[28] de Avila L.D., Daudt C.E. (1997). Indução da fermentação maloláctica em vinho Gewürztraminer. Cienc. Rural..

[29] Binati R.L. (2015). Avaliação da fermentação maloláctica em vinhos de altitude com bactérias ácido-lácticas autóctones selecionadas. https://repositorio.ufsc.br/bitstream/handle/123456789/135391/334491.pdf?sequence=1&isAllowed=y.

[30] Guillamón J.M., Mas A. (2009). Acetic Acid Bacteria. Biology of Microorganisms on Grapes, in Must and Wine.

[31] De Roos J., Verce M., Aerts M., Vandamme P., De Vuyst L. (2018). Temporal and Spatial Distribution of the Acetic Acid Bacterium Communities throughout the Wooden Casks Used for the Fermentation and Maturation of Lambic Beer Underlines Their Functional Role. Appl. Environ. Microbiol..

[32] Wang S., Li C., Ying W., Wang S., Zhou Y., Wu W., Lei Y. (2024). Addition of Lactic Acid Bacteria Modulates Microbial Community and Promotes the Flavor Profiles of Kombucha. Food Biosci..

[33] Naik V., Kerkar S. (2024). Fermentation assisted functional foods. Futur. Trends Biotechnol..

[34] Di Cagno R., Filannino P., Gobbetti M. (2016). Fermented Foods: Fermented Vegetables and Other Products. Encycl. Food Health.

[35] Kolniak-Ostek J., Oszmiański J., Wojdyło A. (2013). Effect of l-ascorbic acid addition on quality, polyphenolic compounds and antioxidant capacity of cloudy apple juices. Eur. Food Res. Technol..

[36] Kolniak-Ostek J. (2016). Identification and quantification of polyphenolic compounds in ten pear cultivars by UPLC-PDA-Q/TOF-MS. J. Food Compos. Anal..

[37] Chandran A.K., Stach M., Kucharska A.Z., Sokół-Łętowska A., Szumny A., Moreira H., Szyjka A., Barg E., Kolniak-Ostek J. (2025). Comparison of polyphenol and volatile compounds and in vitro antioxidant, anti-inflammatory, antidiabetic, anti-ageing, and anticancer activities of dry tea leaves. LWT-Food Sci. Technol..

[38] Yang F., Chen C., Ni D., Yang Y., Tian J., Li Y., Chen S., Ye X., Wang L. (2023). Effects of Fermentation on Bioactivity and the Composition of Polyphenols Contained in Polyphenol-Rich Foods: A Review. Foods.

[39] Kim H., Hur S., Lim J., Jin K., Yang T.-H., Keehm I.-S., Kim S.W., Kim T., Kim D. (2023). Enhancement of the phenolic compounds and antioxidant activities of Kombucha prepared using specific bacterial and yeast. Food Biosci..

[40] Zhou D.-D., Saimaiti A., Luo M., Huang S.-Y., Xiong R.-G., Shang A., Gan R.-Y., Li H.-B. (2022). Fermentation with Tea Residues Enhances Antioxidant Activities and Polyphenol Contents in Kombucha Beverages. Antioxidants.

[41] Liang Z., Huang Y., Zhang P., Fang Z. (2023). Impact of fermentation on the structure and antioxidant activity of selective phenolic compounds. Food Biosci..

[42] Sharma R., Diwan B., Singh B.P., Kulshrestha S. (2022). Probiotic fermentation of polyphenols: Potential sources of novel functional foods. Food Prod. Process. Nutr..

[43] Liu S., He Y., He W., Song X., Peng Y., Hu X., Bian S., Li Y., Nie S., Yin J. (2024). Exploring the Biogenic Transformation Mechanism of Polyphenols by *Lactobacillus plantarum* NCU137 Fermentation and Its Enhancement of Antioxidant Properties in Wolfberry Juice. J. Agric. Food Chem..

[44] Su J., Fu X., Zhang R., Li X., Li Y., Chu X. (2023). Exploring the Effects of Solid-State Fermentation on Polyphenols in Acanthopanax senticosus Based on Response Surface Methodology and Nontargeted Metabolomics Techniques. J. Food Biochem..

[45] Battikh H., Bakhrouf A., Ammar E. (2012). Antimicrobial effect of Kombucha analogues. LWT-Food Sci. Technol..

[46] Sudarshan K., Aidhen I.S. (2016). Convenient Synthesis of 3-Glycosylated Isocoumarins. Eur. J. Org. Chem..

[47] Angeli L., Populin F., Morozova K., Ding Y., Asma U., Bolchini S., Cebulj A., Busatto N., Costa F., Ferrentino G. (2024). Comparative analysis of antioxidant activity and capacity in apple varieties: Insights from stopped flow DPPH• kinetics, mass spectrometry and electrochemistry. Food Biosci..

[48] Kartelias I.G., Karantonis H.C., Giaouris E., Panagiotakopoulos I., Nasopoulou C. (2023). Kombucha Fermentation of Olympus Mountain Tea (*Sideritis scardica*) Sweetened with Thyme Honey: Physicochemical Analysis and Evaluation of Functional Properties. Foods.

[49] Kartelias I.G., Panagiotakopoulos I., Nasopoulou C., Karantonis H.C. (2024). Evaluating the Effect of Adding Selected Herbs, Spices, and Fruits to Fermented Olympus Mountain Tea (*Sideritis scardica*) Kombucha Sweetened with Thyme Honey: Assessment of Physicochemical and Functional Properties. Beverages.

[50] Kim Y., Keogh J.B., Clifton P.M. (2016). Polyphenols and Glycemic Control. Nutrients.

[51] Rupasinghe H.P.V., Balasuriya N., Wang Y. (2017). Prevention of Type 2 Diabetes by Polyphenols of Fruits. Nutritional Antioxidant Therapies: Treatments and Perspectives.

[52] Zin C.A.J.C.M., Mohamed W.M.I.W., Khan N.A.K., Ishak W.R.W. (2022). Effects of Fruit and Vegetable Polyphenols on the Glycemic Control and Metabolic Parameters in Type 2 Diabetes Mellitus: A Review. Prev. Nutr. Food Sci..

[53] Aryaeian N., Sedehi S.K., Arablou T. (2017). Polyphenols and their effects on diabetes management: A review. Med. J. Islam. Repub. Iran.

[54] Visvanathan R., Williamson G. (2021). Effect of citrus fruit and juice consumption on risk of developing type 2 diabetes: Evidence on polyphenols from epidemiological and intervention studies. Trends Food Sci. Technol..

[55] Chen Y., Qie X., Quan W., Zeng M., Qin F., Chen J., Adhikari B., He Z. (2021). Omnifarious fruit polyphenols: An omnipotent strategy to prevent and intervene diabetes and related complication?. Crit. Rev. Food Sci. Nutr..

[56] Wang B., Liu K., Mi M., Wang J. (2014). Effect of Fruit Juice on Glucose Control and Insulin Sensitivity in Adults: A Meta-Analysis of 12 Randomized Controlled Trials. PLoS ONE.

[57] Murphy M.M., Barrett E.C., Barraj L.M. (2017). 100% Fruit juice and measures of glycemic control and insulin sensitivity: A meta-analysis of randomized controlled trials. FASEB J..

[58] Mendelson C., Sparkes S., Merenstein D.J., Christensen C., Sharma V., Desale S., Auchtung J.M., Kok C.R., Hallen-Adams H.E., Hutkins R. (2023). Kombucha tea as an anti-hyperglycemic agent in humans with diabetes—A randomized controlled pilot investigation. Front. Nutr..

[59] de Miranda J.F., Ruiz L.F., Uekane T.M., Silva K.A., Lima A.R. (2022). Kombucha e Seu Potencial Efeito Antidiabético: Revisão.

[60] Amalia R.I., Fahrudin F.I., Alvi A.S., Intipunya P. (2024). Myths and Potential Benefits of Kombucha as a Functional Food: A Review. Int. J. Res. Innov. Appl. Sci..

[61] Filho A.B.B., Barros L.A.d.O., Ribeiro K.R.d.C., da Silva C.A., Koike B.D.V. (2024). Efficiency of daily kombucha consumption in reducing glycemic levels and hypercholesterolemia. Braz. J. Health Rev..

[62] Xu S., Wang Y., Wang J., Geng W. (2022). Kombucha Reduces Hyperglycemia in Type 2 Diabetes of Mice by Regulating Gut Microbiota and Its Metabolites. Foods.

[63] de Bortoli Beal S., Silva C.A., do Amaral A.P.L., Florz J.A.K., Potuk C.F.W., Adami E.R. (2024). Utilização da kombucha em pacientes diabéticos: Uma revisão de literatura. Rev. Gestao E Secr..

[64] Ramanan M., Sinha S., Sudarshan K., Aidhen I.S., Doble M. (2016). Inhibition of the enzymes in the leukotriene and prostaglandin pathways in inflammation by 3-aryl isocoumarins. Eur. J. Med. Chem..

[65] Wahyanto K.N., Agustini R. (2024). Total Flavonoid Content and In Vitro Anti-Inflammatory Potentials of Kombucha with Enrichment of Butterfly Pea (*Clitoria ternatea*) Flower Extract. J. Pijar Mipa.

