# Supplementary material for: Evaluation of Anti-Inflammatory, Antidiabetic, Antioxidant, and Anticholinergic Activities, as Well as Chemical Composition and Polyphenolic Compounds in Novel SCOBY-Fermented Juices
Source: Molecules. 2025 Apr 27;30(9):1940. doi: 10.3390/molecules30091940 (PMC12073502; doi:10.3390/molecules30091940)
Supplement: Supplementary file 1 [file molecules-30-01940-s001.zip › molecules-3582541-supplementary.pdf]

Table S1. The content of phenolic compounds (mg/100 mL) in apple juice during fermentation (average ± standard deviation; n = 3).

Compound	Apple juice								
	Fresh juice	Day 0	Day 2	Day 4	Day 6	Day 8	Day 10	Day 12	Day 14
Phenolic acids									
Caffeoylhexose	3.2 ± 0.1^{a}	3.2 ± 0.1^{a}	3.0 ± 0.1^{a}	3.0 ± 0.1^{a}	2.7 ± 0.1^{b}	1.8 ± 0.0^{c}	1.5 ± 0.1^{c}	1.4 ± 0.0^{c}	1.5 ± 0.1^{c}
Coumaric acid derivative	36.5 ± 0.8^{a}	36.4 ± 0.5^{a}	31.3 ± 0.4^{b}	30.1 ± 0.8^{b}	29.7 ± 0.5^{b}	16.5 ± 0.7^{c}	17.4 ± 0.6^{c}	28.9 ± 0.9^{b}	31.2 ± 1.0^{b}
Caffeoylhexose	6.7 ± 0.3 ^{ab}	6.6 ± 0.2^{ab}	5.3 ± 0.1^{c}	6.9 ± 0.2^{a}	7.0 ± 0.2^{a}	6.7 ± 0.1^{ab}	5.2 ± 0.2^{c}	5.7 ± 0.1^{b}	6.3 ± 0.1^{b}
Caffeoylhexose	4.6 ± 0.1^{d}	4.4 ± 0.1^{d}	3.1 ± 0.2^{e}	5.4 ± 0.2^{c}	7.7 ± 0.2^{c}	15.2 ± 0.4^{b}	18.7 ± 0.3^{b}	20.9 ± 0.4^{a}	22.1 ± 0.5^{a}
Caffeoylhexose	37.0 ± 1.2^{e}	37.6 ± 0.8^{e}	41.3 ± 0.7^{d}	41.3 ± 1.0^{d}	45.6 ± 0.9^{d}	56.3 ± 0.8^{c}	59.3 ± 1.1^{c}	65.9 ± 1.0^{b}	71.8 ± 1.2^{a}
trans-5-p-Coumaroyloquinic acid	4.9 ± 0.0^{a}	4.9 ± 0.1^{a}	4.2 ± 0.1^{b}	4.0 ± 0.1^{b}	3.8 ± 0.0^{c}	2.8 ± 0.0^{d}	3.3 ± 0.1^{c}	3.4 ± 0.1^{c}	3.6 ± 0.0^{c}
4-Caffeoylquinic acid	281.6 ± 3.6^{a}	299.2 ± 2.6^{a}	291.7 ± 3.1^{a}	176.1 ± 3.2^{c}	160.8 ± 2.8^{d}	174.6 ± 2.5^{c}	182.3 ± 3.0^{bc}	201.0 ± 2.8^{b}	295.4 ± 3.2^{a}
cis-4-Caffeoylquinic acid	10.1 ± 0.5^{a}	9.3 ± 0.2^{ab}	8.8 ± 0.2^{b}	8.1 ± 0.3^{c}	7.0 ± 0.1^{d}	8.0 ± 0.1^{c}	9.0 ± 0.2^{ab}	8.8 ± 0.1^{b}	8.8 ± 0.3^{b}
1-Caffeoylquinic acid	9.5 ± 0.3^{b}	9.3 ± 0.2^{b}	10.9 ± 0.1^{a}	8.8 ± 0.3^{c}	9.0 ± 0.2^{bc}	9.3 ± 0.2^{b}	8.2 ± 0.1^{d}	8.3 ± 0.3^{d}	8.8 ± 0.1^{c}
cis-5-p-Coumaroyloquinic acid	21.3 ± 0.7^{a}	20.6 ± 0.5^{b}	21.4 ± 0.4^{a}	21.2 ± 0.1^{a}	16.2 ± 0.5^{e}	21.6 ± 0.3^{a}	19.0 ± 0.1^{d}	20.1 ± 0.0^{c}	20.8 ± 0.4^{ab}
Sum	415.4 ± 6.9^{b}	431.4 ± 8.5^{b}	421.1 ± 7.9^{b}	305.0 ± 6.5^{e}	289.4 ± 5.2^{f}	312.7 ± 7.4^{e}	323.8 ± 8.3^{d}	364.3 ± 5.9^{c}	470.2 ± 4.8^{a}
Flavanols and procyanidins									
B-type Procyanidin dimer	47.5 ± 2.3^{a}	45.8 ± 0.6^{a}	30.5 ± 0.2^{b}	28.0 ± 0.8^{e}	29.1 ± 0.1^{d}	29.8 ± 0.0^{cd}	29.3 ± 0.2^{d}	30.6 ± 0.3^{b}	30.5 ± 0.3^{b}
B-type Procyanidin trimer	55.1 ± 1.5^{a}	52.4 ± 0.3^{a}	54.9 ± 0.4^{a}	44.5 ± 0.2^{b}	44.4 ± 0.2^{b}	40.4 ± 0.4^{bc}	38.9 ± 0.3^{c}	39.8 ± 0.3^{c}	41.6 ± 0.2^{b}
B-type Procyanidin tetramer	45.2 ± 0.9^{a}	44.7 ± 0.2^{a}	46.5 ± 0.4^{a}	34.6 ± 0.2^{e}	38.6 ± 0.3^{b}	35.7 ± 0.2^{d}	32.3 ± 0.3^{f}	35.3 ± 0.4^{d}	36.9 ± 0.3^{c}
B-type Procyanidin trimer	123.2 ± 2.2^{a}	118.5 ± 0.5^{a}	54.5 ± 0.5^{d}	84.5 ± 0.4^{c}	90.0 ± 0.6^{b}	89.8 ± 0.8^{b}	89.3 ± 0.5^{b}	92.2 ± 0.7^{b}	93.6 ± 0.6^{b}
A-type Procyanidin tetramer	345.9 ± 2.6^{a}	343.00 ± 2.5^{a}	317.6 ± 3.2^{b}	293.2 ± 2.9^{c}	181.0 ± 2.5^{d}	151.8 ± 1.9^{e}	139.1 ± 1.5^{f}	118.2 ± 1.8^{g}	106.9 ± 1.5^{h}
B-type Procyanidin tetramer	33.6 ± 0.3^{b}	31.4 ± 0.2^{c}	38.7 ± 0.3^{a}	30.2 ± 0.2^{cd}	31.9 ± 0.1^{c}	28.7 ± 0.2^{d}	31.4 ± 0.3^{c}	33.7 ± 0.3^{b}	36.1 ± 0.2^{a}
(+)-Catechin	37.9 ± 0.5^{f}	37.3 ± 0.5^{f}	52.8 ± 0.4^{e}	59.4 ± 0.5^{d}	70.4 ± 0.6^{c}	80.5 ± 0.5^{b}	89.2 ± 0.7^{a}	90.3 ± 0.6^{a}	91.1 ± 0.8^{a}
A-type Procyanidin trimer	280.9 ± 1.5^{a}	279.5 ± 1.0^{a}	228.1 ± 0.9^{b}	180.1 ± 1.2^{c}	157.7 ± 1.1^{e}	188.0 ± 1.0^{c}	183.0 ± 1.2^{c}	177.7 ± 1.3^{d}	178.6 ± 1.1^{d}
B-type Procyanidin tetramer	42.6 ± 0.6^{a}	41.7 ± 0.3^{a}	37.0 ± 0.4^{b}	26.5 ± 0.2^{d}	24.4 ± 0.2^{d}	26.3 ± 0.2^{d}	31.4 ± 0.3^{c}	31.3 ± 0.3^{c}	32.9 ± 0.2^{c}

B-type Procyanidin dimer	46.9 ± 0.3^{b}	46.5 ± 0.3^{b}	50.0 ± 0.2^{a}	31.1 ± 0.3^{d}	32.5 ± 0.1^{d}	30.4 ± 0.3^{d}	40.5 ± 0.4^{c}	40.8 ± 0.2^{c}	44.9 ± 0.3^{b}
(-)-Epicatechin	48.5 ± 0.5^{b}	45.1 ± 0.4^{c}	45.9 ± 0.5^{c}	46.8 ± 0.2^{bc}	48.1 ± 0.3^{b}	48.8 ± 0.4^{b}	50.8 ± 0.2^{ab}	52.2 ± 0.3^{a}	54.0 ± 0.3^{a}
Sum	1107.3 ± 10.2^{a}	1085.9 ± 9.8^{b}	956.5 ± 9.7^{c}	858.9 ± 10.1^{d}	747.9 ± 7.4^{e}	750.0 ± 5.8^{e}	755.1 ± 10.1^{e}	742.2 ± 8.5^{e}	747.0 ± 8.7^{e}
Flavonols									
Quercetin-3-O-galactoside	1.6 ± 0.1^{a}	1.4 ± 0.1^{a}	1.4 ± 0.1^{a}	1.3 ± 0.1^{a}	1.2 ± 0.1^{ab}	1.0 ± 0.1^{b}	1.0 ± 0.0^{b}	1.0 ± 0.1^{b}	0.9 ± 0.0^{b}
Quercetin-3-O-glucoside	2.5 ± 0.0^{a}	2.4 ± 0.1^{a}	2.3 ± 0.1^{a}	2.3 ± 0.0^{a}	2.2 ± 0.1^{a}	2.2 ± 0.2^{a}	2.2 ± 0.1^{a}	2.1 ± 0.2^{ab}	2.0 ± 0.1^{b}
Quercetin-3-O-xyloside	1.2 ± 0.1^{a}	0.9 ± 0.0^{a}	0.9 ± 0.1^{a}	0.9 ± 0.0^{a}	0.8 ± 0.0^{ab}	0.8 ± 0.0^{ab}	0.8 ± 0.0^{ab}	0.8 ± 0.0^{ab}	0.7 ± 0.0^{b}
Quercetin-3-O-arabinoside	2.1 ± 0.1^{a}	2.0 ± 0.1^{a}	1.9 ± 0.1^{a}	1.8 ± 0.2^{ab}	1.9 ± 0.2^{a}	1.7 ± 0.1^{b}	1.7 ± 0.2^{b}	1.7 ± 0.1^{b}	1.5 ± 0.2^{c}
Quercetin-3-O-rhamnoside	6.5 ± 0.2^{a}	6.5 ± 0.2^{a}	6.4 ± 0.1^{a}	6.5 ± 0.2^{a}	6.5 ± 0.1^{a}	6.5 ± 0.2^{a}	6.5 ± 0.2 ^{a}	6.5 ± 0.1^{a}	6.5 ± 0.2^{a}
Quercetin-3-O-xyloside	4.0 ± 0.1^{a}	3.9 ± 0.1^{a}	3.9 ± 0.1^{a}	3.8 ± 0.2^{ab}	3.7 ± 0.1^{b}	3.7 ± 0.0^{b}	3.7 ± 0.2^{b}	3.6 ± 0.3^{b}	3.7 ± 0.1^{b}
Sum	17.9 ± 0.5^{a}	17.1 ± 0.9^{b}	16.7 ± 0.6^{b}	16.5 ± 0.4^{bc}	16.3 ± 0.5^{bc}	16.0 ± 0.7^{c}	15.8 ± 0.5^{cd}	15.6 ± 0.9^{d}	15.3 ± 0.6^{e}
Dihydrochalcones									
Phloretin 2'-O-xyloglucoside	4.1 ± 0.1^{a}	3.9 ± 0.1^{a}	3.9 ± 0.2^{a}	3.9 ± 0.1^{a}	3.8 ± 0.3^{a}	3.9 ± 0.2^{a}	3.9 ± 0.2^{a}	3.8 ± 0.1^{a}	3.8 ± 0.2^{a}
Phloretin 2'-O-glucoside	24.6 ± 0.4^{d}	23.9 ± 0.5^{d}	25.2 ± 0.6^{c}	25.6 ± 0.5^{c}	25.9 ± 0.3^{c}	27.3 ± 0.4^{b}	28.1 ± 0.5^{a}	28.8 ± 0.6^{a}	27.4 ± 0.7^{b}
Sum	28.7 ± 0.7 ^{c}	27.9 ± 0.5^{d}	29.1 ± 0.5^{c}	29.4 ± 0.7^{c}	29.7 ± 0.6^{bc}	31.1 ± 0.4^{b}	32.0 ± 0.5^{a}	32.5 ± 0.7^{a}	31.1 ± 0.8^{b}
**TOTAL**	**1569.3 ± 9.9**^{a}	**1562.3 ± 10.1**^{a}	**1424.3 ± 10.5**^{b}	**1209.8 ± 11.0**^{c}	**1083.3 ± 10.4**^{e}	**1109.7 ± 9.8**^{de}	**1126.7 ± 10.2**^{d}	**1154.7 ± 10.4**^{d}	**1263.6 ± 11.0**^{c}

Means of three separate analyses ± standard deviation. Duncan's test reveals significant differences ($p < 0.05$) between values in the same rows with different letters (a-g).

Table S2. The content of phenolic compounds (mg/100 mL) in pear juice during fermentation (average ± standard deviation; n = 3).

Compound	Pear juice								
	Fresh juice	Day 0	Day 2	Day 4	Day 6	Day 8	Day 10	Day 12	Day 14
Phenolic acids									
Coffeoylhexose	5.7 ± 0.1^{c}	5.7 ± 0.1^{c}	6.2 ± 0.1^{c}	8.5 ± 0.3^{b}	8.3 ± 0.3^{b}	5.9 ± 0.2^{c}	8.4 ± 0.3^{b}	8.0 ± 0.2^{b}	9.2 ± 0.3^{a}
Coffeoylhexose	5.4 ± 0.1^{e}	5.3 ± 0.0^{e}	6.0 ± 0.1^{d}	7.5 ± 0.2^{c}	8.4 ± 0.3^{b}	7.4 ± 0.2^{c}	10.4 ± 0.3^{a}	9.1 ± 0.2^{b}	10.5 ± 0.2^{a}
Quinic acid	3.0 ± 0.2^{e}	3.0 ± 0.1^{e}	3.7 ± 0.0^{d}	4.1 ± 0.1^{d}	4.7 ± 0.1^{c}	5.2 ± 0.1^{c}	5.5 ± 0.1^{c}	6.4 ± 0.3^{b}	7.3 ± 0.2^{a}
Coffeoylhexose	2.3 ± 0.1^{d}	2.2 ± 0.0^{d}	2.4 ± 0.0^{d}	3.0 ± 0.1^{c}	3.5 ± 0.2^{b}	2.5 ± 0.1^{d}	3.6 ± 0.2^{b}	3.1 ± 0.1^{c}	4.0 ± 0.1^{a}
Caffeoyl N- tryptophan	7.9 ± 0.3^{b}	7.8 ± 0.4^{b}	7.8 ± 0.1^{b}	8.4 ± 0.2^{b}	7.4 ± 0.2^{c}	6.6 ± 0.3^{d}	9.0 ± 0.2^{a}	7.9 ± 0.2^{b}	9.5 ± 0.3^{a}
Syryngic acid galactoside	0.5 ± 0.0^{d}	0.5 ± 0.0^{d}	0.5 ± 0.0^{d}	0.5 ± 0.0^{d}	1.1 ± 0.1^{b}	0.8 ± 0.0^{c}	1.1 ± 0.1^{b}	0.9 ± 0.0^{b}	1.6 ± 0.1^{a}
p-Coumaric acid	0.2 ± 0.0^{c}	0.2 ± 0.0^{c}	0.2 ± 0.0^{c}	0.2 ± 0.0^{c}	0.3 ± 0.0^{c}	0.7 ± 0.0^{b}	1.0 ± 0.0^{b}	1.1 ± 0.1^{b}	1.6 ± 0.2^{a}
3-Caffeoylquinic acid	0.9 ± 0.0^{c}	0.8 ± 0.0^{d}	0.8 ± 0.0^{d}	0.9 ± 0.0^{d}	0.7 ± 0.0^{d}	1.4 ± 0.1^{c}	1.8 ± 0.2^{b}	1.4 ± 0.1^{c}	2.8 ± 0.1^{a}
cis-3-Caffeoylquinic acid	1.3 ± 0.1^{b}	1.2 ± 0.0^{b}	1.2 ± 0.0^{b}	0.9 ± 0.0^{c}	1.2 ± 0.1^{b}	1.0 ± 0.1^{c}	1.6 ± 0.1^{b}	1.4 ± 0.0^{b}	2.8 ± 0.2^{a}
cis-4-p-Coumaroylquinic acid	0.4 ± 0.0 ^{cd}	0.3 ± 0.0^{d}	0.3 ± 0.0^{d}	0.1 ± 0.0^{e}	0.5 ± 0.0^{c}	0.3 ± 0.0^{d}	1.0 ± 0.0^{b}	0.8 ± 0.1^{b}	1.7 ± 0.1^{a}
5-Caffeoylquinic acid	0.3 ± 0.0^{cd}	0.3 ± 0.0^{cd}	0.4 ± 0.0^{c}	0.5 ± 0.0^{c}	0.5 ± 0.0^{c}	0.8 ± 0.0^{b}	1.0 ± 0.1^{a}	1.0 ± 0.0^{a}	0.7 ± 0.0^{b}
Ferulic acid hexoside	1.3 ± 0.1^{a}	1.2 ± 0.0^{a}	1.1 ± 0.0^{a}	1.0 ± 0.0^{a}	1.0 ± 0.1^{a}	0.5 ± 0.0^{b}	0.4 ± 0.0^{b}	0.2 ± 0.0^{c}	0.2 ± 0.0^{c}
Syryngic acid glucoside	0.9 ± 0.0^{cd}	0.9 ± 0.0^{cd}	0.8 ± 0.1^{d}	1.0 ± 0.1^{c}	1.6 ± 0.1^{b}	1.1 ± 0.1^{c}	1.5 ± 0.1^{b}	1.0 ± 0.0^{c}	2.1 ± 0.2^{a}
Sinapic acid hexoside	0.9 ± 0.1^{d}	0.8 ± 0.0^{d}	0.9 ± 0.1^{d}	1.1 ± 0.1^{d}	2.1 ± 0.2^{b}	1.5 ± 0.2^{c}	2.0 ± 0.2^{b}	1.4 ± 0.1^{c}	3.1 ± 0.2^{a}
4-Caffeoylquinic acid	142.3 ± 3.2^{cd}	140.0 ± 5.2^{d}	125.3 ± 4.4^{e}	144.1 ± 4.9^{c}	148.3 ± 6.1^{c}	155.4 ± 4.8^{bc}	159.8 ± 5.0^{b}	161.4 ± 5.1^{b}	168.8 ± 4.9^{a}
Caffeoyl-l-malic acid	8.4 ± 0.3^{a}	8.3 ± 0.5^{a}	8.1 ± 0.6^{a}	8.2 ± 0.2^{a}	4.2 ± 0.2^{b}	2.6 ± 0.1^{d}	3.1 ± 0.1^{c}	2.2 ± 0.1^{d}	3.6 ± 0.2^{c}
p-Coumaroylhexose	1.8 ± 0.2^{a}	1.8 ± 0.2^{a}	1.4 ± 0.1^{b}	0.7 ± 0.0^{c}	0.7 ± 0.0^{c}	0.0 ± 0.0^{d}	0.0 ± 0.0^{d}	0.0 ± 0.0^{d}	0.0 ± 0.0^{d}
1-Caffeoylquinic acid	3.0 ± 0.1^{e}	2.9 ± 0.1^{e}	2.9 ± 0.2^{e}	3.6 ± 0.1^{d}	4.8 ± 0.1^{b}	3.6 ± 0.1^{d}	5.3 ± 0.2^{b}	4.1 ± 0.2^{c}	7.2 ± 0.2^{a}
3-p-Coumaroylquinic acid	0.4 ± 0.0^{c}	0.4 ± 0.1^{c}	0.4 ± 0.0^{c}	0.4 ± 0.0^{c}	0.4 ± 0.0^{c}	0.5 ± 0.0^{b}	0.6 ± 0.0^{b}	0.4 ± 0.0^{c}	1.1 ± 0.1^{a}
di-O-Caffeoylquinic acid	1.1 ± 0.1^{b}	1.0 ± 0.0^{b}	1.2 ± 0.0^{b}	1.1 ± 0.0^{b}	0.7 ± 0.0^{d}	0.8 ± 0.0^{c}	0.9 ± 0.0^{bc}	0.7 ± 0.0^{d}	1.5 ± 0.1^{a}

Sum	188.0 ± 4.8^{d}	184.6 ± 5.6^{d}	171.3 ± 5.0^{e}	195.7 ± 5.2^{c}	200.3 ± 6.1^{c}	198.5 ± 3.8^{c}	217.9 ± 4.4^{b}	212.3 ± 5.3^{b}	239.2 ± 3.2^{a}
Flavanols and procyanidins									
B-type Procyanidin dimer	15.5 ± 1.0^{a}	15.2 ± 1.2^{a}	10.3 ± 0.9^{b}	8.6 ± 0.5^{c}	8.5 ± 0.3^{c}	7.4 ± 0.2^{d}	6.6 ± 0.3^{d}	6.5 ± 0.2^{d}	6.3 ± 0.5^{d}
B-type Procyanidin dimer	13.4 ± 0.6^{a}	13.3 ± 0.4^{a}	12.7 ± 1.1^{a}	11.8 ± 0.2^{b}	11.7 ± 0.2^{b}	11.7 ± 0.3^{b}	11.3 ± 0.3^{bc}	10.6 ± 0.5^{c}	10.6 ± 0.4^{c}
B-type Procyanidin tetramer	11.9 ± 0.3^{a}	11.7 ± 0.2^{a}	9.0 ± 0.7^{b}	7.5 ± 0.1^{c}	5.9 ± 0.1^{d}	5.5 ± 0.1^{d}	5.1 ± 0.2^{e}	5.0 ± 0.3^{e}	4.8 ± 0.0^{e}
B-type Procyanidin dimer	6.9 ± 0.3^{a}	6.8 ± 0.3^{a}	6.4 ± 0.5^{a}	6.7 ± 0.2^{a}	6.8 ± 0.1^{a}	6.4 ± 0.1^{a}	6.7 ± 0.1^{a}	6.9 ± 0.3^{a}	6.5 ± 0.2^{a}
B-type Procyanidin dimer	42.3 ± 1.9^{ab}	40.6 ± 2.8^{b}	43.4 ± 1.2^{a}	43.7 ± 0.6^{a}	37.8 ± 0.5^{c}	39.5 ± 0.3^{b}	36.7 ± 0.3^{c}	27.9 ± 0.4^{d}	36.8 ± 0.3^{c}
B-type Procyanidin tetramer	26.1 ± 1.0^{a}	24.0 ± 1.1^{b}	24.1 ± 1.0^{b}	22.6 ± 0.8^{c}	21.5 ± 0.0^{c}	0.0 ± 0.0^{d}	0.0 ± 0.0^{d}	0.0 ± 0.0^{d}	0.0 ± 0.0^{d}
B-type Procyanidin tetramer	10.9 ± 0.5^{a}	10.1 ± 0.5^{a}	10.2 ± 0.4^{a}	10.2 ± 0.1^{a}	10.1 ± 0.2^{a}	10.3 ± 0.1^{a}	10.4 ± 0.2^{a}	10.5 ± 0.2^{a}	10.1 ± 0.3^{a}
B-type Procyanidin tetramer	12.5 ± 0.4^{a}	11.0 ± 0.9^{b}	6.3 ± 0.4^{c}	6.4 ± 0.1^{c}	5.8 ± 0.1^{d}	6.0 ± 0.1^{cd}	5.5 ± 0.1^{e}	5.4 ± 0.1^{e}	5.0 ± 0.1^{f}
B-type procyanidin dimer	11.9 ± 0.5^{f}	10.4 ± 0.2^{f}	8.7 ± 0.6^{f}	18.7 ± 0.3^{e}	30.3 ± 0.3^{c}	25.6 ± 0.3^{d}	35.7 ± 0.3^{b}	27.2 ± 0.4^{c}	44.1 ± 0.3^{a}
B-type Procyanidin trimer	6.6 ± 0.2^{a}	6.4 ± 0.2^{a}	6.7 ± 0.3^{a}	6.6 ± 0.2^{a}	6.5 ± 0.1^{a}	6.3 ± 0.1^{a}	6.4 ± 0.2^{a}	5.6 ± 0.2^{b}	5.4 ± 0.2^{b}
(+)-Catechin	10.5 ± 0.2^{e}	9.9 ± 0.7^{e}	8.3 ± 0.4^{f}	13.0 ± 0.2^{c}	14.0 ± 0.2^{c}	11.2 ± 0.2^{d}	15.3 ± 0.2^{b}	11.7 ± 0.3^{d}	17.9 ± 0.2^{a}
A-type Procyanidin trimer	84.0 ± 1.9^{a}	81.1 ± 2.7^{a}	61.9 ± 0.8^{b}	34.7 ± 0.4^{c}	34.8 ± 0.2^{c}	25.5 ± 0.3^{d}	17.1 ± 0.3^{e}	15.6 ± 0.2^{f}	9.6 ± 0.1^{g}
Double-charged pentameric procyanidin	1.9 ± 0.1^{a}	1.4 ± 0.3^{b}	1.3 ± 0.2^{b}	1.8 ± 0.1^{a}	1.8 ± 0.1^{a}	2.0 ± 0.1^{a}	1.1 ± 0.2^{b}	1.0 ± 0.0^{b}	0.6 ± 0.0^{c}
Double-charged heptameric procyanidin	4.6 ± 0.2^{ab}	3.8 ± 0.1^{b}	3.7 ± 0.1^{b}	5.1 ± 0.2^{a}	5.3 ± 0.1^{a}	3.0 ± 0.1^{c}	3.5 ± 0.0^{b}	1.7 ± 0.1^{d}	1.9 ± 0.1^{d}
B-type Procyanidin trimer	3.2 ± 0.1^{b}	2.7 ± 0.1^{c}	2.7 ± 0.1^{c}	3.3 ± 0.1^{b}	4.0 ± 0.1^{a}	2.0 ± 0.1^{d}	2.0 ± 0.1^{d}	2.1 ± 0.0^{d}	2.1 ± 0.1^{d}
(-)-Epicatechin	2.5 ± 0.1^{a}	0.0 ± 0.0^{d}	0.1 ± 0.0^{d}	0.6 ± 0.0^{c}	0.8 ± 0.0^{c}	1.0 ± 0.0^{b}	1.1 ± 0.1^{b}	1.3 ± 0.1^{b}	2.5 ± 0.1^{a}
Sum	264.7 ± 5.3^{a}	248.3 ± 4.8^{b}	215.9 ± 1.5^{c}	201.4 ± 2.1^{d}	205.7 ± 1.8^{d}	163.2 ± 1.9^{e}	164.3 ± 1.5^{e}	138.9 ± 1.4^{f}	164.2 ± 1.8^{e}
Flavonols									
Kaempferol hexoside-dideoxyhexoside	0.6 ± 0.0^{a}	0.5 ± 0.0^{a}	0.4 ± 0.0^{ab}	0.5 ± 0.0^{a}	0.4 ± 0.0^{ab}	0.5 ± 0.0^{a}	0.6 ± 0.0^{a}	0.4 ± 0.0^{ab}	0.3 ± 0.0^{b}
Quercetin 3-O-rutinoside	0.8 ± 0.0^{cd}	0.7 ± 0.0^{d}	0.7 ± 0.0^{d}	1.0 ± 0.1^{c}	0.9 ± 0.0^{c}	1.1 ± 0.1^{c}	1.9 ± 0.1^{a}	1.7 ± 0.1^{b}	2.0 ± 0.1^{a}
Quercetin 3-O-galactoside	1.3 ± 0.1^{a}	1.1 ± 0.1^{a}	1.1 ± 0.1^{a}	1.0 ± 0.0^{a}	0.8 ± 0.0^{ab}	0.6 ± 0.0^{c}	0.6 ± 0.0^{c}	0.5 ± 0.0^{cd}	0.4 ± 0.0^{d}
Quercetin 3-O-glucoside	0.6 ± 0.0^{a}	0.4 ± 0.0^{a}	0.4 ± 0.0^{a}	0.4 ± 0.0^{a}	0.3 ± 0.0^{b}	0.3 ± 0.0^{b}	0.3 ± 0.0^{b}	0.3 ± 0.0^{b}	0.3 ± 0.0^{b}

Isorhamnetin 3-O-rhamnosyl-hexoside	1.5 ± 0.1^{b}	1.3 ± 0.1^{c}	1.2 ± 0.1^{c}	1.3 ± 0.1^{c}	2.1 ± 0.1^{a}	1.1 ± 0.1^{c}	1.7 ± 0.1^{b}	1.4 ± 0.1^{c}	2.9 ± 0.2^{a}
Quercetin-acylated-hexoside	2.1 ± 0.1^{a}	1.9 ± 0.1^{b}	2.0 ± 0.1^{ab}	2.2 ± 0.1^{a}	1.7 ± 0.1^{c}	1.7 ± 0.1^{c}	2.3 ± 0.0^{a}	2.0 ± 0.2^{ab}	2.4 ± 0.2^{a}
Isorhamnetin 3-O-rhamnosyl-hexoside	2.1 ± 0.0^{a}	1.8 ± 0.1^{b}	1.8 ± 0.1^{b}	2.0 ± 0.1^{a}	1.6 ± 0.1^{c}	1.5 ± 0.1^{c}	2.0 ± 0.1^{a}	1.8 ± 0.1^{b}	2.1 ± 0.1^{a}
Isorhamnetin 3-O-galactoside	1.5 ± 0.0^{a}	1.5 ± 0.1^{a}	1.4 ± 0.1^{a}	1.4 ± 0.0^{a}	0.7 ± 0.0^{b}	0.7 ± 0.0^{b}	0.6 ± 0.0^{b}	0.5 ± 0.0^{b}	0.3 ± 0.0^{c}
Kaempferol 3-O-glucoside	1.8 ± 0.1^{b}	1.8 ± 0.0^{b}	2.0 ± 0.1^{a}	2.0 ± 0.1^{a}	1.5 ± 0.1^{c}	1.3 ± 0.1^{c}	0.8 ± 0.0^{d}	0.4 ± 0.0^{e}	0.4 ± 0.0^{e}
Isorhamnetin 3-O-glucoside	0.3 ± 0.0^{b}	0.1 ± 0.0^{a}	0.0 ± 0.0^{c}	0.0 ± 0.0^{c}	0.0 ± 0.0^{c}	0.0 ± 0.0^{c}	0.0 ± 0.0^{c}	0.0 ± 0.0^{c}	0.0 ± 0.0^{c}
Kaempferol 3-O-6-acetylated-glucoside	1.1 ± 0.1^{b}	0.9 ± 0.0^{bc}	0.9 ± 0.0^{bc}	1.0 ± 0.0^{b}	0.8 ± 0.0^{c}	0.7 ± 0.0^{c}	1.2 ± 0.1^{b}	0.8 ± 0.0^{c}	1.5 ± 0.1^{a}
Isorhamnetin-acylated-hexoside	3.6 ± 0.1^{b}	3.3 ± 0.1^{c}	3.3 ± 0.1^{c}	3.7 ± 0.1^{b}	3.1 ± 0.1^{d}	2.8 ± 0.1^{e}	4.0 ± 0.2^{a}	3.5 ± 0.2^{b}	3.9 ± 0.1^{a}
Sum	17.3 ± 0.9^{a}	15.1 ± 0.5^{c}	15.2 ± 0.3^{c}	16.3 ± 0.2^{b}	13.8 ± 0.3^{d}	12.2 ± 0.3^{e}	16.0 ± 0.3^{b}	13.5 ± 0.3^{d}	16.5 ± 0.2^{b}
Flavones									
Apigenin pentoside	4.1 ± 0.1^{a}	4.1 ± 0.2^{a}	1.8 ± 0.1^{c}	2.5 ± 0.2^{b}	1.4 ± 0.1^{d}	1.2 ± 0.1^{d}	1.0 ± 0.1^{de}	0.9 ± 0.1^{e}	0.9 ± 0.0^{e}
Hydroquinones									
Arbutin	40.9 ± 1.8^{e}	40.8 ± 2.8^{e}	45.5 ± 2.3^{e}	59.7 ± 2.6^{d}	67.8 ± 2.7^{d}	76.1 ± 3.0^{c}	130.7 ± 3.5^{b}	129.8 ± 3.4^{b}	155.9 ± 2.9^{a}
**TOTAL**	**515.0 ± 5.2^{b}**	**492.9 ± 8.9^{b}**	**449.5 ± 7.5^{d}**	**475.7 ± 8.9^{c}**	**489.00 ± 9.0^{bc}**	**451.3 ± 10.2^{d}**	**530.00 ± 5.8^{a}**	**495.4 ± 7.3^{b}**	**576.7 ± 10.5^{a}**

Means of three separate analyses ± standard deviation. Duncan's test reveals significant differences ($p < 0.05$) between values in the same rows with different letters (a-g).

Table S3. The content [%] of individual polyphenolic compounds in pear juice in relation to their corresponding polyphenol groups during fermentation

Compound		Fresh juice	Day 0	Day 2	Day 4	Day 6	Day 8	Day 10	Day 12	Day 14
Phenolic acids										
Coffeoylhexose		3.0	3.1	3.6	4.3	4.1	3.0	3.9	3.8	3.9
Coffeoylhexose		2.9	2.9	3.5	3.8	4.2	3.7	4.8	4.3	4.4
Quinic acid		1.6	1.6	2.2	2.1	2.4	2.6	2.5	3.0	3.1
Coffeoylhexose		1.2	1.2	1.4	1.5	1.8	1.3	1.7	1.5	1.7
Caffeoyl N- tryptophan		4.2	4.2	4.6	4.3	3.7	3.3	4.1	3.7	4.0
Syryngic acid galactoside		0.3	0.3	0.3	0.3	0.6	0.4	0.5	0.4	0.7
p-Coumaric acid		0.1	0.1	0.1	0.1	0.2	0.4	0.5	0.5	0.7
3-Caffeoylquinic acid		0.5	0.4	0.5	0.5	0.4	0.7	0.8	0.7	1.2
cis-3-Caffeoylquinic acid		0.7	0.7	0.7	0.5	0.6	0.5	0.7	0.7	1.2
cis-4-p-Coumaroylquinic acid		0.2	0.2	0.2	0.1	0.3	0.2	0.5	0.4	0.7
5-Caffeoylquinic acid		0.2	0.2	0.2	0.3	0.3	0.4	0.5	0.5	0.3
Ferulic acid hexoside		0.7	0.7	0.6	0.5	0.5	0.3	0.2	0.1	0.1
Syryngic acid glucoside		0.5	0.5	0.5	0.5	0.8	0.6	0.7	0.5	0.9
Sinapic acid hexoside		0.5	0.4	0.5	0.6	1.1	0.8	0.9	0.7	1.3
4-Caffeoylquinic acid		75.7	75.8	73.2	73.6	74.0	78.3	73.3	76.0	70.6
Caffeoyl-l-malic acid		4.5	4.5	4.7	4.2	2.1	1.3	1.4	1.0	1.5
p-Coumaroylhexose		1.0	1.0	0.8	0.4	0.4	0.0	0.0	0.0	0.0
1-Caffeoylquinic acid		1.6	1.6	1.7	1.8	2.4	1.8	2.4	1.9	3.0
3-p-Coumaroylquinic acid		0.2	0.2	0.2	0.2	0.2	0.3	0.3	0.2	0.5
di-O-Caffeoylquinic acid		0.6	0.5	0.7	0.6	0.4	0.4	0.4	0.3	0.6
	Sum	100	100.0	100.0	100.0	100.0	100.0	100.0	100.0	100.0
Flavanols and procyanidins										
B-type Procyanidin dimer		5.9	6.1	4.8	4.3	4.1	4.5	4.0	4.7	3.8
B-type Procyanidin dimer		5.1	5.4	5.9	5.9	5.7	7.2	6.9	7.6	6.5
B-type Procyanidin tetramer		4.5	4.7	4.2	3.7	2.9	3.4	3.1	3.6	2.9
B-type Procyanidin dimer		2.6	2.7	3.0	3.3	3.3	3.9	4.1	5.0	4.0
B-type Procyanidin dimer		16	16.4	20.1	21.7	18.4	24.2	22.3	20.1	22.4
B-type Procyanidin tetramer		9.9	9.7	11.2	11.2	10.5	0	0	0	0
B-type Procyanidin tetramer		4.1	4.1	4.7	5.1	4.9	6.3	6.3	7.6	6.2
B-type Procyanidin tetramer		4.7	4.4	2.9	3.2	2.8	3.7	3.4	3.9	3.1

B-type procyanidin dimer		4.5	4.2	4.0	9.3	14.7	15.7	21.7	19.6	26.9
B-type Procyanidin trimer		2.5	2.6	3.1	3.3	3.2	3.9	3.9	4.0	3.3
(+)-Catechin		4.0	4.0	3.8	6.5	6.8	6.9	9.3	8.4	10.9
A-type Procyanidin trimer		31.7	32.7	28.7	17.2	16.9	15.6	10.4	11.2	5.9
Double-charged pentameric procyanidin		0.7	0.6	0.6	0.9	0.9	1.2	0.7	0.7	0.4
Double-charged heptameric procyanidin		1.7	1.5	1.7	2.5	2.6	1.8	2.1	1.2	1.2
B-type Procyanidin trimer		1.2	1.1	1.3	1.6	1.9	1.2	1.2	1.5	1.3
(-)-Epicatechin		0.9	0	0.1	0.3	0.4	0.6	0.7	0.9	1.5
	Sum	100	100	100	100	100	100	100	100	100
Flavonols										
Kaempferol hexoside-dideoxyhexoside		3.5	3.3	2.6	3.1	2.9	4.1	3.8	3.0	1.8
Quercetin 3-O-rutinoside		4.6	4.6	4.6	6.1	6.5	9.0	11.9	12.6	12.1
Quercetin 3-O-galactoside		7.5	7.3	7.2	6.1	5.8	4.9	3.8	3.7	2.4
Quercetin 3-O-glucoside		3.5	2.7	2.6	2.5	2.2	2.5	1.9	2.2	1.8
Isorhamnetin 3-O-rhamnosyl-hexoside		8.7	8.6	7.9	8.0	15.2	9.0	10.6	10.4	17.6
Quercetin-acylated-hexoside		12.1	12.6	13.2	13.5	12.3	13.9	14.4	14.8	14.6
Isorhamnetin 3-O-rhamnosyl-hexoside		12.1	11.9	11.8	12.3	11.6	12.3	12.5	13.3	12.7
Isorhamnetin 3-O-galactoside		8.7	9.9	9.2	8.6	5.1	5.7	3.8	3.7	1.8
Kaempferol 3-O-glucoside		10.4	11.9	13.2	12.3	10.9	10.7	5.0	3.0	2.4
Isorhamnetin 3-O-glucoside		1.7	0.7	0.3	0.3	0.2	0.0	0.0	0.0	0.0
Kaempferol 3-O-6-acetylated-glucoside		6.4	6.0	5.9	6.1	5.8	5.7	7.5	5.9	9.1
Isorhamnetin-acylated-hexoside		20.8	21.9	21.7	22.7	22.5	23.0	25.0	25.9	23.6
	Sum	100	100	100	100	100	100	100	100	100
Flavones										
Apigenin pentoside		100	100	100	100	100	100	100	100	100
Hydroquinones										
Arbutin		100	100	100	100	100	100	100	100	100

Table S4. The content [%] of individual polyphenolic compounds in pear juice in relation to total phenolic content during fermentation

Compound		Fresh juice	Day 0	Day 2	Day 4	Day 6	Day 8	Day 10	Day 12	Day 14
Phenolic acids										
Coffeoylhexose		1.1	1.2	1.4	1.8	1.7	1.3	1.6	1.6	1.6
Coffeoylhexose		1.1	1.1	1.3	1.6	1.7	1.6	2.0	1.8	1.8
Quinic acid		0.6	0.6	0.8	0.9	1.0	1.2	1.0	1.3	1.3
Coffeoylhexose		0.5	0.5	0.5	0.6	0.7	0.6	0.7	0.6	0.7
Caffeoyl N- tryptophan		1.5	1.6	1.7	1.8	1.5	1.5	1.7	1.6	1.7
Syryngic acid galactoside		0.1	0.1	0.1	0.1	0.2	0.2	0.2	0.2	0.3
p-Coumaric acid		0.0	0.0	0.0	0.0	0.1	0.2	0.2	0.2	0.3
3-Caffeoylquinic acid		0.2	0.2	0.2	0.2	0.1	0.3	0.3	0.3	0.5
cis-3-Caffeoylquinic acid		0.3	0.2	0.3	0.2	0.3	0.2	0.3	0.3	0.5
cis-4-p-Coumaroylquinic acid		0.1	0.1	0.1	0.0	0.1	0.1	0.2	0.2	0.3
5-Caffeoylquinic acid		0.1	0.1	0.1	0.1	0.1	0.2	0.2	0.2	0.1
Ferulic acid hexoside		0.3	0.2	0.2	0.2	0.2	0.1	0.1	0.0	0.0
Syryngic acid glucoside		0.2	0.2	0.2	0.2	0.3	0.2	0.3	0.2	0.4
Sinapic acid hexoside		0.2	0.2	0.2	0.2	0.4	0.3	0.4	0.3	0.5
4-Caffeoylquinic acid		27.6	28.4	27.9	30.3	30.3	34.4	30.2	32.6	29.3
Caffeoyl-l-malic acid		1.6	1.7	1.8	1.7	0.9	0.6	0.6	0.4	0.6
p-Coumaroylhexose		0.4	0.4	0.3	0.2	0.1	0.0	0.0	0.0	0.0
1-Caffeoylquinic acid		0.6	0.6	0.7	0.8	1.0	0.8	1.0	0.8	1.3
3-p-Coumaroylquinic acid		0.1	0.1	0.1	0.1	0.1	0.1	0.1	0.1	0.2
di-O-Caffeoylquinic acid		0.2	0.2	0.3	0.2	0.1	0.2	0.2	0.1	0.3
	Sum	36.5	37.5	38.1	41.1	41.0	44.0	41.1	42.9	41.5
Flavanols and procyanidins										
B-type Procyanidin dimer		3.0	3.1	2.3	1.8	1.7	1.6	1.3	1.3	1.1
B-type Procyanidin dimer		2.6	2.7	2.8	2.5	2.4	2.6	2.1	2.1	1.8
B-type Procyanidin tetramer		2.3	2.4	2.0	1.6	1.2	1.2	1.0	1.0	0.8
B-type Procyanidin dimer		1.3	1.4	1.4	1.4	1.4	1.4	1.3	1.4	1.1
B-type Procyanidin dimer		8.2	8.2	9.7	9.2	7.7	8.8	6.9	5.6	6.4
B-type Procyanidin tetramer		5.1	4.9	5.4	4.8	4.4	0.0	0.0	0.0	0.0
B-type Procyanidin tetramer		2.1	2.1	2.3	2.1	2.1	2.3	2.0	2.1	1.8

B-type Procyanidin tetramer	2.4	2.2	1.4	1.4	1.2	1.3	1.0	1.1	0.9
B-type procyanidin dimer	2.3	2.1	1.9	3.9	6.2	5.7	6.7	5.5	7.7
B-type Procyanidin trimer	1.3	1.3	1.5	1.4	1.3	1.4	1.2	1.1	0.9
(+)-Catechin	2.0	2.0	1.9	2.7	2.9	2.5	2.9	2.4	3.1
A-type Procyanidin trimer	16.3	16.5	13.8	7.3	7.1	5.7	3.2	3.2	1.7
Double-charged pentameric procyanidin	0.4	0.3	0.3	0.4	0.4	0.4	0.2	0.2	0.1
Double-charged heptameric procyanidin	0.9	0.8	0.8	1.1	1.1	0.7	0.7	0.3	0.3
B-type Procyanidin trimer	0.6	0.6	0.6	0.7	0.8	0.4	0.4	0.4	0.4
(-)-Epicatechin	0.5	0.0	0.0	0.1	0.2	0.2	0.2	0.3	0.4
Sum	51.4	50.4	48.0	42.3	42.1	36.2	31.0	28.0	28.5
Flavonols									
Kaempferol hexoside-dideoxyhexoside	0.1	0.1	0.1	0.1	0.1	0.1	0.1	0.1	0.1
Quercetin 3-O-rutinoside	0.2	0.1	0.2	0.2	0.2	0.2	0.4	0.3	0.4
Quercetin 3-O-galactoside	0.3	0.2	0.2	0.2	0.2	0.1	0.1	0.1	0.1
Quercetin 3-O-glucoside	0.1	0.1	0.1	0.1	0.1	0.1	0.1	0.1	0.1
Isorhamnetin 3-O-rhamnosyl-hexoside	0.3	0.3	0.3	0.3	0.4	0.2	0.3	0.3	0.5
Quercetin-acylated-hexoside	0.4	0.4	0.4	0.5	0.4	0.4	0.4	0.4	0.4
Isorhamnetin 3-O-rhamnosyl-hexoside	0.4	0.4	0.4	0.4	0.3	0.3	0.4	0.4	0.4
Isorhamnetin 3-O-galactoside	0.3	0.3	0.3	0.3	0.1	0.2	0.1	0.1	0.1
Kaempferol 3-O-glucoside	0.4	0.4	0.4	0.4	0.3	0.3	0.2	0.1	0.1
Isorhamnetin 3-O-glucoside	0.1	0.0	0.0	0.0	0.0	0.0	0.0	0.0	0.0
Kaempferol 3-O-6-acetylated-glucoside	0.2	0.2	0.2	0.2	0.2	0.2	0.2	0.2	0.3
Isorhamnetin-acylated-hexoside	0.7	0.7	0.7	0.8	0.6	0.6	0.8	0.7	0.7
Sum	3.4	3.1	3.4	3.4	2.8	2.7	3.0	2.7	2.9
Flavones									
Apigenin pentoside	0.8	0.8	0.4	0.5	0.3	0.3	0.2	0.2	0.2
Hydroquinones									
Arbutin	7.9	8.3	10.1	12.6	13.9	16.9	24.7	26.2	27.0
**TOTAL**	100.0	100.0	100.0	100.0	100.0	100.0	100.0	100.0	100.0

Table S5. The content [%] of individual polyphenolic compounds in apple juice in relation to their corresponding polyphenol groups during fermentation

Phenolic acids	Fresh juice	Day 0	Day 2	Day 4	Day 6	Day 8	Day 10	Day 12	Day 14
Quinic acid	1.6	1.5	1.5	1.7	1.7	1.7	1.6	1.5	1.1
Caffeoylhexose	0.8	0.7	0.7	1.0	0.9	0.6	0.5	0.4	0.3
Coumaric acid derivative	8.6	8.3	7.3	9.7	10.1	5.2	5.3	7.8	6.6
Caffeoylhexose	1.6	1.5	1.2	2.2	2.4	2.1	1.6	1.5	1.3
Caffeoylhexose	1.1	1.0	0.7	1.7	2.6	4.8	5.7	5.7	4.6
Caffeoylhexose	8.8	8.6	9.7	13.3	15.5	17.7	18.0	17.8	15.1
trans-5-p-Coumaroyloquinic acid	1.2	1.1	1.0	1.3	1.3	0.9	1.0	0.9	0.8
4-Caffeoylquinic acid	66.7	68.3	68.2	56.7	54.6	54.9	55.4	54.4	62.1
cis-4-Caffeoylquinic acid	2.4	2.1	2.1	2.6	2.4	2.5	2.7	2.4	1.9
1-Caffeoylquinic acid	2.3	2.1	2.5	2.8	3.1	2.9	2.5	2.2	1.9
cis-5-p-Coumaroyloquinic acid	5.0	4.7	5.0	6.8	5.5	6.8	5.8	5.4	4.4
Sum	100.0	100.0	100.0	100.0	100.0	100.0	100.0	100.0	100.0
Flavanols and procyanidins									
B-type Procyanidin dimer	4.3	4.2	3.2	3.3	3.9	4.0	3.9	4.1	4.1
B-type Procyanidin trimer	5.0	4.8	5.7	5.2	5.9	5.4	5.2	5.4	5.6
B-type Procyanidin tetramer	4.1	4.1	4.9	4.0	5.2	4.8	4.3	4.8	4.9
B-type Procyanidin trimer	11.1	10.9	5.7	9.8	12.0	12.0	11.8	12.4	12.5
A-type Procyanidin tetramer	31.2	31.6	33.2	34.1	24.2	20.2	18.4	15.9	14.3
B-type Procyanidin tetramer	3.0	2.9	4.0	3.5	4.3	3.8	4.2	4.5	4.8
(+)-Catechin	3.4	3.4	5.5	6.9	9.4	10.7	11.8	12.2	12.2
A-type Procyanidin trimer	25.4	25.7	23.8	21.0	21.1	25.1	24.2	23.9	23.9
B-type Procyanidin tetramer	3.8	3.8	3.9	3.1	3.3	3.5	4.2	4.2	4.4
B-type Procyanidin dimer	4.2	4.3	5.2	3.6	4.3	4.1	5.4	5.5	6.0
(-)-Epicatechin	4.4	4.2	4.8	5.4	6.4	6.5	6.7	7.0	7.2
Sum	100.0	100.0	100.0	100.0	100.0	100.0	100.0	100.0	100.0
Flavonols									
Quercetin-3-O-galactoside	8.9	8.2	8.4	7.9	7.4	6.3	6.3	6.4	5.9
Quercetin-3-O-glucoside	14.0	14.0	13.8	13.9	13.5	13.8	13.9	13.5	13.1
Quercetin-3-O-xyloside	6.7	5.3	5.4	5.5	4.9	5.0	5.1	5.1	4.6

Quercetin-3-O-arabinoside		11.7	11.7	11.4	10.9	11.7	10.6	10.8	10.9	9.8
Quercetin-3-O-rhamnoside		36.3	38.0	38.3	39.4	39.9	40.6	41.1	41.7	42.5
Quercetin-3-O-xyloside		22.3	22.8	23.4	23.0	22.7	23.1	23.4	23.1	24.2
	Sum	100.0	100.0	100.0	100.0	100.0	100.0	100.0	100.0	100.0
Dihydrochalcones										
Phloretin 2'-O-xyloglucoside		14.3	14.0	13.4	13.3	12.8	12.5	12.2	11.7	12.2
Phloretin 2'-O-glucoside		85.7	85.7	86.6	87.1	87.2	87.8	87.8	88.6	88.1
	Sum	100.0	100.0	100.0	100.0	100.0	100.0	100.0	100.0	100.0

Table S6. The content [%] of individual polyphenolic compounds in apple juice in relation to total phenolic content during fermentation

Phenolic acids	Fresh juice	Day 0	Day 2	Day 4	Day 6	Day 8	Day 10	Day 12	Day 14
Quinic acid	0,4	0,4	0,5	0,4	0,5	0,5	0,5	0,5	0,4
Caffeoylhexose	0,2	0,2	0,2	0,2	0,2	0,2	0,1	0,1	0,1
Coumaric acid derivative	2,3	2,3	2,2	2,5	2,7	1,5	1,5	2,5	2,5
Caffeoylhexose	0,4	0,4	0,4	0,6	0,6	0,6	0,5	0,5	0,5
Caffeoylhexose	0,3	0,3	0,2	0,4	0,7	1,4	1,7	1,8	1,7
Caffeoylhexose	2,3	2,4	2,9	3,4	4,2	5,0	5,2	5,7	5,7
trans-5-p-Coumaroyloquinic acid	0,3	0,3	0,3	0,3	0,3	0,3	0,3	0,3	0,3
4-Caffeoylquinic acid	17,9	19,1	20,4	14,5	14,8	15,7	16,1	17,3	23,3
cis-4-Caffeoylquinic acid	0,6	0,6	0,6	0,7	0,6	0,7	0,8	0,8	0,7
1-Caffeoylquinic acid	0,6	0,6	0,8	0,7	0,8	0,8	0,7	0,7	0,7
cis-5-p-Coumaroyloquinic acid	1,4	1,3	1,5	1,7	1,5	1,9	1,7	1,7	1,6
Sum	26,8	27,9	29,9	25,5	27,1	28,5	29,1	31,9	37,5
Flavanols and procyanidins	0,0	0,0	0,0	0,0	0,0	0,0	0,0	0,0	0,0
B-type Procyanidin dimer	3,0	2,9	2,1	2,3	2,7	2,7	2,6	2,6	2,4
B-type Procyanidin trimer	3,5	3,3	3,8	3,7	4,1	3,6	3,4	3,4	3,3
B-type Procyanidin tetramer	2,9	2,8	3,3	2,8	3,5	3,2	2,9	3,0	2,9
B-type Procyanidin trimer	7,8	7,6	3,8	7,0	8,3	8,1	7,9	7,9	7,4
A-type Procyanidin tetramer	21,9	21,9	22,2	24,1	16,6	13,6	12,3	10,2	8,4
B-type Procyanidin tetramer	2,1	2,0	2,7	2,5	2,9	2,6	2,8	2,9	2,8
(+)-Catechin	2,4	2,4	3,7	4,9	6,5	7,2	7,9	7,8	7,2
A-type Procyanidin trimer	17,8	17,8	16,0	14,8	14,5	16,9	16,2	15,3	14,1
B-type Procyanidin tetramer	2,7	2,7	2,6	2,2	2,2	2,4	2,8	2,7	2,6
B-type Procyanidin dimer	3,0	3,0	3,5	2,6	3,0	2,7	3,6	3,5	3,5
(-)-Epicatechin	3,1	2,9	3,2	3,9	4,4	4,4	4,5	4,5	4,3
Sum	70,3	69,2	66,9	70,7	68,7	67,3	66,7	64,0	58,9
Flavonols	0,0	0,0	0,0	0,0	0,0	0,0	0,0	0,0	0,0
Quercetin-3-O-galactoside	0,1	0,1	0,1	0,1	0,1	0,1	0,1	0,1	0,1
Quercetin-3-O-glucoside	0,2	0,2	0,2	0,2	0,2	0,2	0,2	0,2	0,2
Quercetin-3-O-xyloside	0,1	0,1	0,1	0,1	0,1	0,1	0,1	0,1	0,1
Quercetin-3-O-arabinoside	0,1	0,1	0,1	0,1	0,2	0,2	0,2	0,1	0,1

Quercetin-3-O-rhamnoside	0,4	0,4	0,4	0,5	0,6	0,6	0,6	0,6	0,5
Quercetin-3-O-xyloside	0,3	0,2	0,3	0,3	0,3	0,3	0,3	0,3	0,3
Sum	1,1	1,1	1,2	1,4	1,5	1,4	1,4	1,3	1,2
Dihydrochalcones	0,0	0,0	0,0	0,0	0,0	0,0	0,0	0,0	0,0
Phloretin 2'-O-xyloglucoside	0,3	0,2	0,3	0,3	0,3	0,3	0,3	0,3	0,3
Phloretin 2'-O-glucoside	1,6	1,5	1,8	2,1	2,4	2,4	2,5	2,5	2,2
Sum	1,8	1,8	2,0	2,4	2,7	2,8	2,8	2,8	2,5
**TOTAL**	100	100	100	100	100	100	100	100	100

Table S7. The rate of the biotransformation reaction [mg/h] of polyphenolic compounds in pear juice during fermentation

	V [mg/h]							
Compound	Day 0	Day 2	Day 4	Day 6	Day 8	Day 10	Day 12	Day 14
Phenolic acids								
Coffeoylhexose	0	10.4×10^{-3}	47.9×10^{-3}	4.2×10^{-3}	50.0×10^{-3}	52.1×10^{-3}	8.3×10^{-3}	25.0×10^{-3}
Coffeoylhexose	0	14.6×10^{-3}	31.3×10^{-3}	18.8×10^{-3}	20.8×10^{-3}	62.5×10^{-3}	27.1×10^{-3}	29.2×10^{-3}
Quinic acid	0	14.6×10^{-3}	8.3×10^{-3}	12.5×10^{-3}	10.4×10^{-3}	6.3×10^{-3}	18.8×10^{-3}	18.8×10^{-3}
Coffeoylhexose	0	4.2×10^{-3}	12.5×10^{-3}	10.4×10^{-3}	20.8×10^{-3}	22.9×10^{-3}	10.4×10^{-3}	18.8×10^{-3}
Caffeoyl N- tryptophan	0	0	12.5×10^{-3}	20.8×10^{-3}	16.7×10^{-3}	50.0×10^{-3}	22.9×10^{-3}	33.3×10^{-3}
Syryngic acid galactoside	0	0	0	12.5×10^{-3}	6.3×10^{-3}	6.3×10^{-3}	4.2×10^{-3}	14.6×10^{-3}
p-Coumaric acid	0	0	0	2.1×10^{-3}	8.3×10^{-3}	6.3×10^{-3}	2.1×10^{-3}	10.4×10^{-3}
3-Caffeoylquinic acid	0	0	2.1×10^{-3}	4.2×10^{-3}	14.6×10^{-3}	8.3×10^{-3}	8.3×10^{-3}	29.2×10^{-3}
cis-3-Caffeoylquinic acid	0	0	6.3×10^{-3}	6.3×10^{-3}	4.2×10^{-3}	12.5×10^{-3}	4.2×10^{-3}	29.2×10^{-3}
cis-4-p-Coumaroylquinic acid	0	0	4.2×10^{-3}	8.3×10^{-3}	4.2×10^{-3}	14.6×10^{-3}	4.2×10^{-3}	18.8×10^{-3}
5-Caffeoylquinic acid	0	2.1×10^{-3}	2.1×10^{-3}	0	6.3×10^{-3}	4.2×10^{-3}	0	6.3×10^{-3}
Ferulic acid hexoside	0	2.1×10^{-3}	2.1×10^{-3}	0	10.4×10^{-3}	2.1×10^{-3}	4.2×10^{-3}	0
Syryngic acid glucoside	0	2.1×10^{-3}	4.2×10^{-3}	12.5×10^{-3}	10.4×10^{-3}	8.3×10^{-3}	10.4×10^{-3}	22.9×10^{-3}
Sinapic acid hexoside	0	2.1×10^{-3}	4.2×10^{-3}	20.8×10^{-3}	12.5×10^{-3}	10.4×10^{-3}	12.5×10^{-3}	35.4×10^{-3}
4-Caffeoylquinic acid	0	306.3×10^{-3}	391.7×10^{-3}	87.5×10^{-3}	147.9×10^{-3}	91.7×10^{-3}	33.3×10^{-3}	154.2×10^{-3}
Caffeoyl-l-malic acid	0	4.2×10^{-3}	2.1×10^{-3}	83.3×10^{-3}	33.3×10^{-3}	10.4×10^{-3}	18.8×10^{-3}	29.2×10^{-3}
p-Coumaroylhexose	0	8.3×10^{-3}	14.6×10^{-3}	0	14.6×10^{-3}	0	0	0
1-Caffeoylquinic acid	0	0	14.7×10^{-3}	25.0×10^{-3}	25.0×10^{-3}	35.4×10^{-3}	25.0×10^{-3}	64.6×10^{-3}
3-p-Coumaroylquinic acid	0	0	0	0	2.1×10^{-3}	2.1×10^{-3}	4.2×10^{-3}	14.6×10^{-3}
di-O-Caffeoylquinic acid	0	4.2×10^{-3}	2.1×10^{-3}	8.3×10^{-3}	2.1×10^{-3}	2.1×10^{-3}	4.2×10^{-3}	16.7×10^{-3}
Sum		277.1×10^{-3}	508.3×10^{-3}	95.8×10^{-3}	37.5×10^{-3}	404.2×10^{-3}	116.7×10^{-3}	560.4×10^{-3}
Flavanols and procyanidins								
B-type Procyanidin dimer	0	102.1×10^{-3}	35.4×10^{-3}	2.1×10^{-3}	22.9×10^{-3}	16.7×10^{-3}	2.1×10^{-3}	4.2×10^{-3}
B-type Procyanidin dimer	0	12.5×10^{-3}	18.8×10^{-3}	2.1×10^{-3}	0	8.3×10^{-3}	14.6×10^{-3}	0
B-type Procyanidin tetramer	0	56.3×10^{-3}	31.3×10^{-3}	33.3×10^{-3}	8.3×10^{-3}	8.3×10^{-3}	2.1×10^{-3}	4.2×10^{-3}
B-type Procyanidin dimer	0	8.3×10^{-3}	6.3×10^{-3}	2.1×10^{-3}	8.3×10^{-3}	6.3×10^{-3}	4.2×10^{-3}	8.3×10^{-3}
B-type Procyanidin dimer	0	58.3×10^{-3}	6.3×10^{-3}	122.9×10^{-3}	35.4×10^{-3}	58.3×10^{-3}	183.3×10^{-3}	185.4×10^{-3}
B-type Procyanidin tetramer	0	2.1×10^{-3}	31.3×10^{-3}	22.9×10^{-3}	447.9×10^{-3}	0	0	0

B-type Procyanidin tetramer		0	2.1×10^{-3}	0	2.1×10^{-3}	4.2×10^{-3}	2.1×10^{-3}	2.1×10^{-3}	8.3×10^{-3}
B-type Procyanidin tetramer		0	97.9×10^{-3}	2.1×10^{-3}	12.5×10^{-3}	4.2×10^{-3}	10.4×10^{-3}	2.1×10^{-3}	8.3×10^{-3}
B-type procyanidin dimer		0	35.4×10^{-3}	208.3×10^{-3}	241.7×10^{-3}	97.9×10^{-3}	210.4×10^{-3}	177.1×10^{-3}	352.1×10^{-3}
B-type Procyanidin trimer		0	6.3×10^{-3}	2.1×10^{-3}	2.1×10^{-3}	4.2×10^{-3}	2.1×10^{-3}	16.7×10^{-3}	4.2×10^{-3}
(+)-Catechin		0	33.3×10^{-3}	97.9×10^{-3}	20.8×10^{-3}	58.3×10^{-3}	85.4×10^{-3}	75.0×10^{-3}	129.2×10^{-3}
A-type Procyanidin trimer		0	400.0×10^{-3}	566.7×10^{-3}	2.1×10^{-3}	193.8×10^{-3}	175.0×10^{-3}	31.3×10^{-3}	125.0×10^{-3}
Double-charged pentameric procyanidin		0	2.1×10^{-3}	10.4×10^{-3}	0	4.2×10^{-3}	18.8×10^{-3}	2.1×10^{-3}	8.3×10^{-3}
Double-charged heptameric procyanidin		0	2.1×10^{-3}	29.2×10^{-3}	4.2×10^{-3}	47.9×10^{-3}	10.4×10^{-3}	37.5×10^{-3}	4.2×10^{-3}
B-type Procyanidin trimer		0	0	12.5×10^{-3}	14.6×10^{-3}	41.7×10^{-3}	0	2.1×10^{-3}	0
(-)-Epicatechin		0	2.1×10^{-3}	10.4×10^{-3}	4.2×10^{-3}	4.2×10^{-3}	2.1×10^{-3}	4.2×10^{-3}	25.0×10^{-3}
	Sum	0	675.0×10^{-3}	302.1×10^{-3}	89.6×10^{-3}	885.4×10^{-3}	22.9×10^{-3}	529.2×10^{-3}	527.1×10^{-3}
Flavonols									
Kaempferol hexoside-dideoxyhexoside		0	2.1×10^{-3}	2.1×10^{-3}	2.1×10^{-3}	2.1×10^{-3}	2.1×10^{-3}	4.2×10^{-3}	2.1×10^{-3}
Quercetin 3-O-rutinoside		0	0	6.3×10^{-3}	2.1×10^{-3}	4.2×10^{-3}	16.7×10^{-3}	4.2×10^{-3}	6.3×10^{-3}
Quercetin 3-O-galactoside		0	0	2.1×10^{-3}	4.2×10^{-3}	4.2×10^{-3}	0	2.1×10^{-3}	2.1×10^{-3}
Quercetin 3-O-glucoside		0	0	0	2.1×10^{-3}	0	0	0	0
Isorhamnetin 3-O-rhamnosyl-hexoside		0	2.1×10^{-3}	2.1×10^{-3}	16.7×10^{-3}	20.8×10^{-3}	12.5×10^{-3}	6.3×10^{-3}	31.3×10^{-3}
Quercetin-acylated-hexoside		0	2.1×10^{-3}	4.2×10^{-3}	10.4×10^{-3}	0	12.5×10^{-3}	6.3×10^{-3}	8.3×10^{-3}
Isorhamnetin 3-O-rhamnosyl-hexoside		0	0	4.2×10^{-3}	8.3×10^{-3}	2.1×10^{-3}	10.4×10^{-3}	4.2×10^{-3}	6.3×10^{-3}
Isorhamnetin 3-O-galactoside		0	2.1×10^{-3}	0	14.6×10^{-3}	0	2.1×10^{-3}	2.1×10^{-3}	4.2×10^{-3}
Kaempferol 3-O-glucoside		0	4.2×10^{-3}	0	10.4×10^{-3}	4.2×10^{-3}	10.4×10^{-3}	8.3×10^{-3}	0
Isorhamnetin 3-O-glucoside		0	1.3×10^{-3}	0	0.2×10^{-3}	0.6×10^{-3}	0	0	0
Kaempferol 3-O-6-acetylated-glucoside		0	0	2.1×10^{-3}	4.2×10^{-3}	2.1×10^{-3}	10.4×10^{-3}	8.3×10^{-3}	14.6×10^{-3}
Isorhamnetin-acylated-hexoside		0	0	8.3×10^{-3}	12.5×10^{-3}	6.3×10^{-3}	25.0×10^{-3}	10.4×10^{-3}	8.3×10^{-3}
	Sum		2.1×10^{-3}	22.9×10^{-3}	52.1×10^{-3}	33.3×10^{-3}	79.2×10^{-3}	52.1×10^{-3}	62.5×10^{-3}
Flavones									
Apigenin pentoside		0	47.9×10^{-3}	14.6×10^{-3}	22.9×10^{-3}	4.2×10^{-3}	4.2×10^{-3}	2.1×10^{-3}	0
Hydroquinones									
Arbutin		0	97.9×10^{-3}	295.8×10^{-3}	168.8×10^{-3}	172.9×10^{-3}	1137.5×10^{-3}	18.7×10^{-3}	543.8×10^{-3}
	**TOTAL**	0	904.2×10^{-3}	545.8×10^{-3}	277.1×10^{-3}	785.4×10^{-3}	1639.6×10^{-3}	720.8×10^{-3}	1693.8×10^{-3}

Table S8. The rate of the biotransformation reaction [mg/h] of polyphenolic compounds in apple juice during fermentation

		V [mg/h]							
Phenolic acids		Day 0	Day 2	Day 4	Day 6	Day 8	Day 10	Day 12	Day 14
Quinic acid		0	0	22.9 x 10^{-3}	6.3 x 10^{-3}	6.3 x 10^{-3}	0	0	0
Caffeoylhexose		0	4.2 x 10^{-3}	0	6.3 x 10^{-3}	18.8 x 10^{-3}	6.3 x 10^{-3}	2.1 x 10^{-3}	2.1 x 10^{-3}
Coumaric acid derivative		0	106.3 x 10^{-3}	25.0 x 10^{-3}	8.3 x 10^{-3}	275.0 x 10^{-3}	18.8 x 10^{-3}	239.6 x 10^{-3}	47.9 x 10^{-3}
Caffeoylhexose		0	27.1 x 10^{-3}	33.3 x 10^{-3}	2.1 x 10^{-3}	6.3 x 10^{-3}	31.3 x 10^{-3}	10.4 x 10^{-3}	12.5 x 10^{-3}
Caffeoylhexose		0	27.1 x 10^{-3}	47.9 x 10^{-3}	47.9 x 10^{-3}	156.3 x 10^{-3}	72.9 x 10^{-3}	45.8 x 10^{-3}	25.0 x 10^{-3}
Caffeoylhexose		0	77.1 x 10^{-3}	0	89.6 x 10^{-3}	222.9 x 10^{-3}	62.5 x 10^{-3}	137.5 x 10^{-3}	122.9 x 10^{-3}
trans-5-p-Coumaroyloquinic acid		0	14.6 x 10^{-3}	4.2 x 10^{-3}	4.2 x 10^{-3}	20.8 x 10^{-3}	10.4 x 10^{-3}	2.1 x 10^{-3}	4.2 x 10^{-3}
4-Caffeoylquinic acid		0	156.3 x 10^{-3}	2408.3 x 10^{-3}	318.8 x 10^{-3}	287.5 x 10^{-3}	160.4 x 10^{-3}	389.6 x 10^{-3}	1966.7 x 10^{-3}
cis-4-Caffeoylquinic acid		0	10.4 x 10^{-3}	14.6 x 10^{-3}	22.9 x 10^{-3}	20.8 x 10^{-3}	20.8 x 10^{-3}	4.2 x 10^{-3}	0
1-Caffeoylquinic acid		0	33.3 x 10^{-3}	43.8 x 10^{-3}	4.2 x 10^{-3}	6.3 x 10^{-3}	22.9 x 10^{-3}	2.1 x 10^{-3}	10.4 x 10^{-3}
cis-5-p-Coumaroyloquinic acid		0	16.7 x 10^{-3}	4.2 x 10^{-3}	4.2 x 10^{-3}	112.5 x 10^{-3}	54.2 x 10^{-3}	22.9 x 10^{-3}	14.6 x 10^{-3}
	Sum	0	214.6 x 10^{-3}	2441.7 x 10^{-3}	331.3 x 10^{-3}	491.7 x 10^{-3}	231.2 x 10^{-3}	843.8 x 10^{-3}	2206.3 x 10^{-3}
Flavanols and procyanidins									
B-type Procyanidin dimer		0	318.8 x 10^{-3}	52.1 x 10^{-3}	22.9 x 10^{-3}	14.6 x 10^{-3}	10.4 x 10^{-3}	27.1 x 10^{-3}	2.1 x 10^{-3}
B-type Procyanidin trimer		0	52.1 x 10^{-3}	216.7 x 10^{-3}	2.1 x 10^{-3}	83.3 x 10^{-3}	31.3 x 10^{-3}	18.8 x 10^{-3}	37.5 x 10^{-3}
B-type Procyanidin tetramer		0	37.5 x 10^{-3}	247.9 x 10^{-3}	83.3 x 10^{-3}	60.4 x 10^{-3}	70.8 x 10^{-3}	62.5 x 10^{-3}	33.3 x 10^{-3}
B-type Procyanidin trimer		0	1333.3 x 10^{-3}	625.0 x 10^{-3}	114.6 x 10^{-3}	4.2 x 10^{-3}	10.4 x 10^{-3}	60.4 x 10^{-3}	29.2 x 10^{-3}
A-type Procyanidin tetramer		0	529.2 x 10^{-3}	508.3 x 10^{-3}	2337.5 x 10^{-3}	608.3 x 10^{-3}	264.6 x 10^{-3}	435.4 x 10^{-3}	235.4 x 10^{-3}
B-type Procyanidin tetramer		0	152.1 x 10^{-3}	177.1 x 10^{-3}	35.4 x 10^{-3}	66.7 x 10^{-3}	56.3 x 10^{-3}	47.9 x 10^{-3}	50.0 x 10^{-3}
(+)-Catechin		0	322.9 x 10^{-3}	137.5 x 10^{-3}	229.2 x 10^{-3}	210.4 x 10^{-3}	181.3 x 10^{-3}	22.9 x 10^{-3}	16.7 x 10^{-3}
A-type Procyanidin trimer		0	1070.8 x 10^{-3}	1000.0 x 10^{-3}	466.7 x 10^{-3}	631.3 x 10^{-3}	104.2 x 10^{-3}	110.4 x 10^{-3}	18.8 x 10^{-3}
B-type Procyanidin tetramer		0	97.9 x 10^{-3}	218.8 x 10^{-3}	43.8 x 10^{-3}	39.6 x 10^{-3}	106.3 x 10^{-3}	2.1 x 10^{-3}	33.3 x 10^{-3}
B-type Procyanidin dimer		0	72.9 x 10^{-3}	393.8 x 10^{-3}	29.2 x 10^{-3}	43.8 x 10^{-3}	210.4 x 10^{-3}	6.2 x 10^{-3}	85.4 x 10^{-3}
(-)-Epicatechin		0	16.7 x 10^{-3}	18.8 x 10^{-3}	27.1 x 10^{-3}	14.6 x 10^{-3}	41.7 x 10^{-3}	29.2 x 10^{-3}	37.5 x 10^{-3}
	Sum	0	2695.8 x 10^{-3}	2033.3 x 10^{-3}	2312.5 x 10^{-3}	43.8 x 10^{-3}	106.3 x 10^{-3}	268.8 x 10^{-3}	100.0 x 10^{-3}
Flavonols									
Quercetin-3-O-galactoside		0	0	2.1 x 10^{-3}	2.1 x 10^{-3}	4.2 x 10^{-3}	0	0	2.1 x 10^{-3}
Quercetin-3-O-glucoside		0	2.1 x 10^{-3}	0	2.1 x 10^{-3}	0	0	2.1 x 10^{-3}	2.1 x 10^{-3}
Quercetin-3-O-xyloside		0	0	0	2.1 x 10^{-3}	0	0	0	2.1 x 10^{-3}

Quercetin-3-O-arabinoside		0	2.1×10^{-3}	2.1×10^{-3}	2.1×10^{-3}	4.2×10^{-3}	0	0	4.2×10^{-3}
Quercetin-3-O-rhamnoside		0	2.1×10^{-3}	2.1×10^{-3}	0	0	0	0	0
Quercetin-3-O-xyloside		0	0	2.1×10^{-3}	2.1×10^{-3}	0	0	2.1×10^{-3}	2.1×10^{-3}
	Sum	0	8.3×10^{-3}	4.2×10^{-3}	4.2×10^{-3}	6.3×10^{-3}	4.2×10^{-3}	4.2×10^{-3}	6.2×10^{-3}
Dihydrochalcones									
Phloretin 2'-O-xyloglucoside		0	0	0	2.1×10^{-3}	2.1×10^{-3}	0	2.1×10^{-3}	0
Phloretin 2'-O-glucoside		0	27.1×10^{-3}	8.3×10^{-3}	6.2×10^{-3}	29.2×10^{-3}	16.7×10^{-3}	14.6×10^{-3}	29.2×10^{-3}
	Sum	0	25.0×10^{-3}	6.2×10^{-3}	6.3×10^{-3}	29.2×10^{-3}	18.8×10^{-3}	10.4×10^{-3}	29.2×10^{-3}
	**TOTAL**	0	2893.8×10^{-3}	4472.9×10^{-3}	2641.7×10^{-3}	556.2×10^{-3}	354.2×10^{-3}	583.3×10^{-3}	2268.8×10^{-3}
